# Will predicted positive effects of climate change be enough to reverse declines of the regionally Endangered Natterjack toad in Ireland?

**DOI:** 10.1002/ece3.7362

**Published:** 2021-03-11

**Authors:** Marina Reyne, Natasha E. McGowan, Jason Flanagan, Paul Nolan, Aurélie Aubry, Mark Emmerson, Ferdia Marnell, Neil Reid

**Affiliations:** ^1^ School of Biological Sciences Queen's University Belfast Belfast UK; ^2^ Irish Centre for High End Computing (ICHEC) Dublin Ireland; ^3^ Agri‐Food and Biosciences (AFBI) Hillsborough UK; ^4^ Institute of Global Food Security (IGFS) Belfast UK; ^5^ National Parks & Wildlife Service (NPWS) Dublin Ireland

**Keywords:** amphibia, bioclim, *Epidalea calamita*, fecundity, GLMM, Maxent, range expansion, SDM, spawning, species distribution model

## Abstract

The global amphibian crisis is driven by a range of stressors including disease, habitat loss, and environmental contamination. The role of climate change remains poorly studied and is likely to influence environmental suitability, ranges, reproduction, and phenology. This study aimed to characterize the bioclimatic‐habitat niche space of the Natterjack toad (*Epidalea calamita*) throughout its European range and to assess the impact of climate on the toad's environmental suitability and breeding behavior in Ireland, where declines in recent decades have resulted in it being regionally Red‐Listed as Endangered. To address these questions, we first identified which climate variables best predict the current bioclimatic niche, fecundity (number of eggs deposit), and phenology. We then used future climate projections for two time periods (2041–2060 and 2061–2080) and two greenhouse gas emission scenarios (RCP 4.5 and RCP 8.5) to predict how the species range, fecundity, and phenology would change. The European range of the species was found to be limited by winter temperatures while its bioclimatic niche varied markedly throughout its range. Species distribution models suggested projected climate change will increase environmental suitability for the species throughout its range, including Ireland, but most notably in Scandinavia and the Baltic. Fecundity in Ireland was greatest during the cool temperatures of spring and after wet winters associated with ephemeral breeding pool availability. Warm, dry summers in the preceding year influenced fecundity the following spring indicative of carryover effects. Initiation of spawning was driven by spring temperatures, not rainfall. Projections suggested future climate change may increase fecundity in Ireland while spawning may commence earlier throughout the 21st century especially under a high greenhouse gas emission scenario (RCP 8.5). Despite recent range contraction and population declines due to habitat deterioration, the Natterjack toad, if subject to a suitable species conservation strategy, has the potential to be a climate change winner, notwithstanding unpredictable habitat and land‐use change, sea‐level rise inducing coastal erosion, changes in invertebrate prey abundance, and disease.

## Graphical abstract

In this study, we assessed the impact of climate change on the Natterjack toad's environmental suitability, fecundity and spawning behavior. Our results suggested projected climate change will increase environmental suitability for the species throughout its distribution range and may increase fecundity while spawning may commence earlier. Despite recent range contraction and population declines due to habitat deterioration, the Natterjack toad, if subject to a suitable species conservation strategy, has the potential to be a climate change winner.

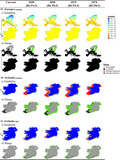

## 1 INTRODUCTION

Amphibians are the most endangered vertebrate group with 41% of species threatened with extinction (IUCN, 2020). Population declines have been detected even in common and widespread species (Adams et al., 2013; Petrovan & Schmidt, 2016; Stuart et al., 2004) as a consequence of ongoing stressors including habitat loss (Cushman, 2006), contamination (Brühl et al., 2013; Mann et al., 2009), disease (Berger et al., 1998; Lips, 1999; Martel et al., 2013), invasive species (Johnson et al., 2011), and illegal harvest and trade (Chan et al., 2014; Schlaepfer et al., 2005). Recent studies suggest that climate change poses an additional serious threat to amphibian populations (e.g., Bombi & D'amen, 2009; Carey & Alexander, 2003; Pounds et al., 2006), directly impacting species behavior and phenology (Semlitsch & Wilbur, 1988), availability of suitable habitat (McMenamin et al., 2008), or by interacting with other factors such as disease (Bosch et al., 2007; Laurance, 2008; Pounds et al., 2006). However, evidence that climate change is directly causing amphibian declines and extinction is weak and controversial (Carey & Alexander, 2003; Li et al., 2013; McCallum, 2005; Rohr et al., 2008). Understanding the role of climate in population dynamics and the potential impact of future climate change on population viability and extinction risk of endangered species is crucial for implementing effective species management strategies (Shoo et al., 2011).

Amphibia are ectotherms, and all aspects of their physiology, behavior, and life history are strongly dependent on weather and climate, especially for temperate species exposed to clearly defined seasons. Temperature impacts their mechanism of gas exchange (Wood & Glass, 1991), metabolic rate (White et al., 2006), immune function (Raffel et al., 2006), and phenology like timing of breeding (Beebee, 1995) and duration of hibernation (Jørgensen, 1986). Activity and breeding migrations are often positively correlated with precipitation (Gibbsons & Bennett, 1974; Smith & Skelcher, 2019). Decreased precipitation and ambient moisture can alter pond hydroperiods, resulting in early or rapid pond desiccation (McMenamin et al., 2008), consequently altering larval development (Reading & Clarke, 1999). Climate change is, therefore, likely to have a significant impact on growth, body condition, reproduction, fecundity, and recruitment, that is, population dynamics and trajectory, of amphibians.

Global mean surface temperature has increased by approximately 0.8°C over the last century and is likely to continue to increase throughout the 21st century by between 2.6 and 4.1°C, calculated based on different greenhouse gas (GHG) emission scenarios (Sherwood et al., 2020). Climate change is generally expected to lead to more variable and intense precipitation with longer periods of drought between precipitation events (IPCC, 2014). Distribution of suitable habitats for a wide variety of species may change by the end of the 21st century, resulting in increased extinction risk, especially for those that are range restricted (Gibson et al., 2010; Marini et al., 2010; Penman et al., 2009; Thomas et al., 2004). Amphibians are likely to be particularly sensitive to climate change given the high proportion of declining populations, dependence on temperature and humidity, high sensitivity to stressors, and low ability to disperse (Blaustein et al., 2001; Carey & Alexander, 2003). Climate change is likely to cause major shifts in spatial patterns of amphibian diversity, resulting in range contraction and expansion (Duan et al., 2016; Zank et al., 2014). Range shifts are the most common response to climate change (Parmesan & Yohe, 2003; Root et al., 2003) and a species' ability to track its suitable bioclimatic envelope will be essential for survival (Sunday et al., 2014).

The Natterjack toad (*Epidalea calamita*) is widespread throughout Europe, ranging from Iberia to the Baltic (Gasc et al., 1997). The species is often associated with scrubby, open habitat on sandy substrates or dry heath with shallow seasonally ephemeral ponds (Beebee & Griffiths, 2000). In some regions of its range, Ireland, for example, the Natterjack toad is regionally Red‐Listed as Endangered due to a 50%–60% range contraction since the 1970s, driven by loss of aquatic and terrestrial habitats, for example, drainage and agricultural intensification, and deterioration of habitat quality, for example, reed encroachment of ponds and undergrazing of terrestrial habitats around ponds, leading to rank vegetation and poor foraging conditions (King et al., 2011). In Ireland, fecundity (numbers of egg strings deposited annually) has also declined at most metapopulations (Reyne et al., 2019) causing concern that population size is declining. The role of climate in changes in range, fecundity, and phenology is unknown.

This study aimed to quantify the impact of climate change on the Natterjack toad throughout its European range and assess changes in a focal range edge population (Ireland). The main objectives were to (a) characterize its bioclimatic‐habitat niche throughout its range, including Ireland, (b) use species distribution models at different spatial and temporal extents to predict the potential impact of projected climate change on environmental suitability and, potentially, suitable range for the species, and (c) model fecundity and initiation of spawning, projecting potential climate change effects on reproduction. Our goal was to predict the impact of climate change on a range edge population regionally Red‐Listed as Endangered to inform species conservation management.

## 2 METHODS

### 2.1 Species records and spawning

**FIGURE 1:**
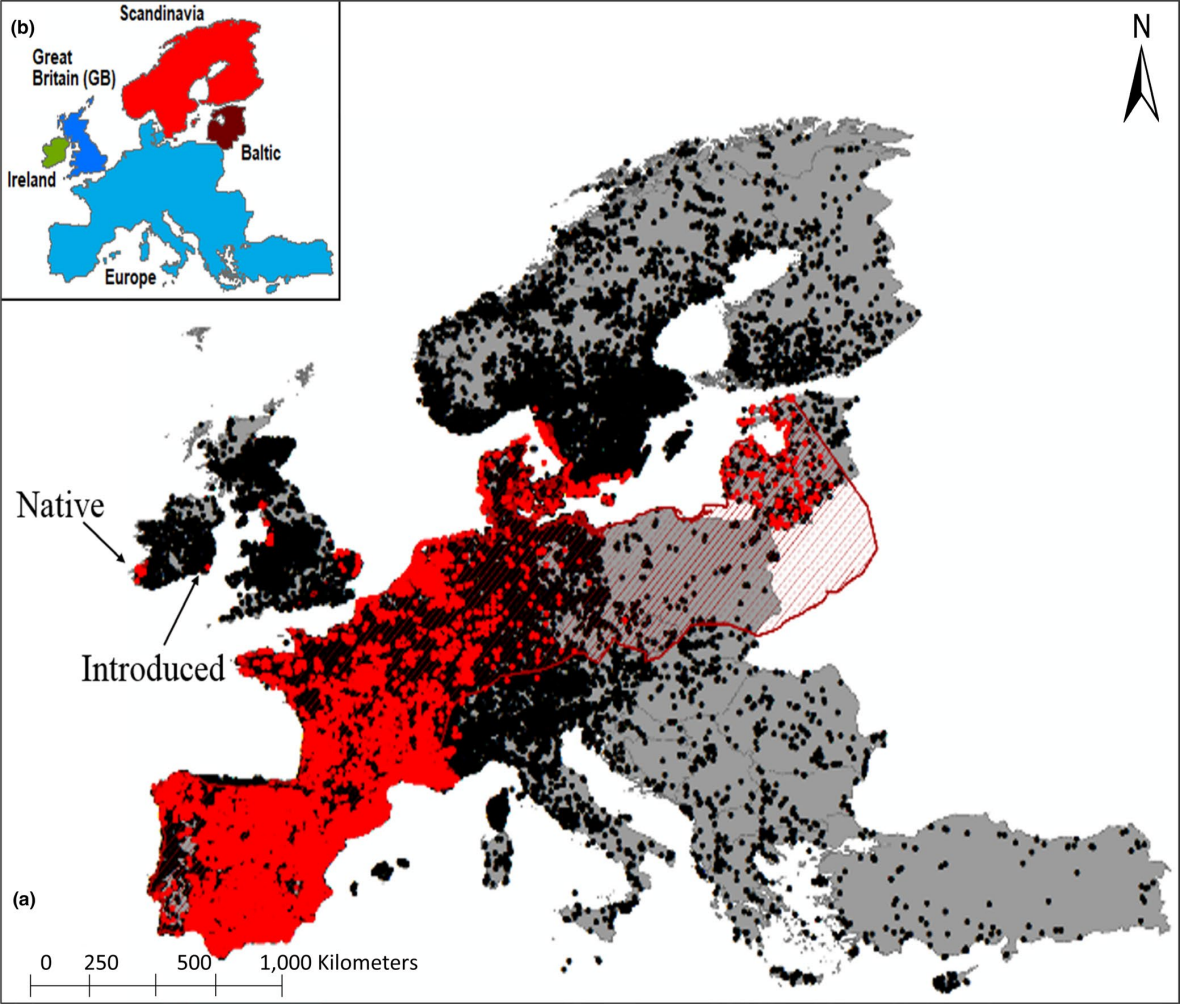
(a) European distribution of Natterjack toad records (red dots *n* = 37,062) overlaid with the IUCN species range polygon (red hatching) and underlaid with GBIF records for 84 other Amphibian species recorded throughout Europe (black dots *n* = 470,245). The Natterjack toad is highly range restricted in Ireland with its native range in the southwest and an introduced population in the southeast (labels). (b) European region names used in analysis for orientation

A total of 470,245 species records for all 84 amphibian species known to occur in Europe, including 37,062 Natterjack toad (*E. calamita*) records, were downloaded from the Global Biodiversity Information Facility (). No GBIF records of the Natterjack toad were available from Poland, suggesting either no recording effort or that the species, while it occurs there (e.g., Franz et al., 2013), is scarce. Nevertheless, Poland was included in the species IUCN range (Figure 1). The GBIF occurrence data and IUCN expert range maps provide best available data on species distribution range and are suitable for climatic niche modeling (Alhajeri & Fourcade, 2019). Ireland represents the extreme north‐western range edge margin of the Natterjack toad distribution where the species is highly range restricted, represented by seven populations in Co Kerry and one introduced population in Co. Wexford (Figure 1). The species has been monitored intensively by three major projects from: (a) 2004–2006 (Bécart et al., 2007), (b) 2011–2012, and (c) 2016–2018 (Reyne et al., 2019). Thus, the number of ponds that formed annually, the presence/absence of Natterjack toads, their fecundity, and spawning dates were known throughout their range in Ireland with a very high degree of accuracy for the years surveyed. For species distribution modeling, species records were reduced in resolution to match that of the input environmental data (~4 km) in order to minimize spatial autocorrelation, errors, and duplicate records, thus decreasing sample sizes (*n* = 40,861 Natterjack records across 443,030 grid cells in Europe, *n* = 24 records across 7,037 grid cells in Ireland at 2.5° and *n* = 11 records across 5,503 grid cells in Ireland at 4 km).

### 2.2 Climate data

Climate at the extent of Europe was characterized by data downloaded from WorldClim (worldclim.org) at a 2.5°(~4 km) grid cell resolution. Of the nineteen available bioclimatic variables, we selected seven based on their relevance to amphibian biology (Table S1; Figure S1). Toads are ectothermic and hibernate during winter, and thus, mean annual temperature (bio1), the diurnal temperature range (bio2), mean temperature of the warmest quarter (bio10), and mean temperature of the coldest quarter (bio11) were selected as potentially relevant to homeostasis, activity, and hibernation (Jørgensen, 1986; Navas et al., 2008; White et al., 2006). Total precipitation (bio12), precipitation of the wettest quarter (bio17), and precipitation of the driest quarter (bio17) were selected as potentially relevant to breeding pool formation and ephemerality (McMenamin et al., 2008; Smith & Skelcher, 2019). Future climatological projections used the HadGEM2‐ES model (Collins et al. 2011) as it incorporated high levels of climatic complexity. Future projections were downloaded for the mid‐century 2050s (average for 2041–2060) and late century 2070s (averaged for 2061–2080) for two representative concentration pathways (RCPs): an intermediate (RCP4.5) and a high (RCP8.5) greenhouse gas emission scenarios, while “current” conditions were taken as averages from 1976 to 2005 matching the timeframe of the data downloaded from GBIF.

Climate at the extent of Ireland has been simulated using the COSMO‐CLM5 Regional Climate Model (Rockel et al., 2008; Steppeler et al., 2003) by the Irish Centre for High End Computing (ICHEC; ichec.ie). Of the twenty‐two available variables, we selected five on their perceived relevance to amphibian biology (Table S1; Figure S2). In addition to surface temperature (T_S) and rainfall (TOT_PREC), we selected variables not available via WorldClim including soil temperature 54cm below ground level (T_SO_00540mm) as relevant to hibernation, subsurface run‐off (RUNOFF_G), and wind speed < 10 m (WDSPD_10m) as relevant to pool formation, water levels, and pool ephemerality. Gridded climate datasets for Ireland, both historical (1976–2005) and future (2021–2100), were generated at temporal and spatial resolutions of 3 hr and 4 km, respectively. Future predictions were again made based on two greenhouse emission scenarios: intermediate (RCP4.5) and high (RCP8.5) (van Vuuren et al. 2011). For a full description of the model configuration and an overview of validations and projections, see Nolan et al. (2017) and Nolan and Flanagan (2020). It is important to note that the spatial and temporal resolution and timeframes covered by the COSMO‐CLM5 data for Ireland (referred as ICHEC) differed from that of WorldClim for Europe or Ireland.

We used the same five variables (COSMO‐CLM5, ICHEC) to estimate the potential climate change effects on Natterjack toad's reproduction behavior. Values were averaged for the 6 hourly 9 a.m. to 3 p.m. period on each date for which an egg string survey had been performed allowing conditions during each survey day to be quantified (Survey*_t_*). We were interested in seasonal lagged effects and calculated average daily values for the focal Spring*_t_* (March–April–May) of each toad breeding season in the year of survey (*_t_*) and seasons in the preceding year (*_t_*_−1_): Spring*_t_*_−1_, Summer*_t_* (June–July–August), Autumn*_t_*_−1_ (September–October–November), and Winter*_t_*_−1_ (December–January–February). Future climatological projections used the COSMO‐CLM5 ensemble, covered the same daily 6 hourly 9 a.m. to 3 p.m. windows, and were averaged for each season and obtained for the mid‐century 2050s (averaged for 2041–2070) and late century 2070s (averaged for 2071–2100) for both RCP4.5 and RCP8.5.

### 2.3 Habitat data

Habitat data were downloaded for CORINE Land Cover 2018 from the European Environment Agency (EEA 2020; https://land.copernicus.eu/pan‐european/corine‐land‐cover/clc2018) and summarized at a 2.5°(~4 km) grid cell resolution throughout Europe and a 4‐km grid cell resolution throughout Ireland to match the two climate datasets. Individual CORINE land codes were aggregated and collapsed to derive simplified, ecologically relevant habitat classifications (Table S1): coastal habitats, freshwater, grassland, scrub, and sparse vegetation. Habitat categories were selected based on known Natterjack toad habitat preferences (Beebee, 1983). As the species is exclusively coastal in Ireland, we calculated the distance of the centroid of each grid cell from the marine high‐water mark, that is, distance to coast. We performed all spatial analysis in ArcMap 10.7.1 (ESRI).

### 2.4 Niche characterization

The Natterjack toad's core range extends from the Mediterranean coast of Iberia, northward through France, and north and east into Germany and the Netherlands where records become more sporadic (Figure 1) Natterjack toads also occur in the Baltic, along the southern coast of Sweden and in Great Britain in highly isolated populations. Climatic conditions in each of these regions are very different, thus to characterize spatial variation in the Natterjack toad's niche tolerance, WorldClim bioclimatic and CORINE habitat variables were extracted for each species record and analyzed using discriminant function analysis (DFA), fitting region (Ireland, Great Britain, Europe, Scandinavia, and the Baltic) as the grouping variable. For each axis with an Eigenvalue > 1, the median, interquartile range, and 95% confidence intervals of axis scores were plotted using a boxplot and differences tested using a one‐way ANOVA with pairwise least significant difference post hoc tests used between each region.

### 2.5 Species distribution models

Species distribution models (SDMs) were constructed using maximum entropy and the program Maxent 3.4.1 (Phillips et al., 2020). As the Natterjack toad's bioclimatic‐habitat niche varied across Europe, three SDMs were created as follows: (a) at the full extent of Europe using WorldClim climate data, hereafter referred to as the Europe_WorldClim_ model, (b) at the extent of Ireland only using WorldClim climate data, hereafter referred to as the Ireland_WorldClim_ model, and (c) at the extent of Ireland using Ireland‐specific downscaled climate variables, hereafter referred to as the Ireland_ICHEC_ model.

Species records represented presence data. To account for some degree of survey effort across Europe, background points (pseudo‐absences) were not drawn at random from throughout the full model extent, but instead were confined to cells in which any of the 84 amphibian species that are known to occur in Europe had been recorded; that is, we could be confident an observer predisposed to submitting an amphibian record was present in the cell but failed to report a Natterjack toad sighting. Thus, background points more closely approximated true absence data than if randomly selected from throughout the extent of Europe. For models at the extent of Ireland, background points were drawn from throughout the model extent as the Natterjack toad is known not to occur anywhere outside its recorded range with certainty; thus, background points reflected true absences.

Species records were split into model training datasets (75% of records chosen randomly) and test datasets (25% chosen randomly) with four replicate model runs (with bootstrapping) such that every record had a roughly equal chance of being selected once as a test record. Model outputs across the four replicate runs were averaged. To minimize model overfitting, hinge and threshold responses were excluded with only linear and quadratic curves fitted to create smoothed (ecologically plausible) response curves for each input variable. A Jackknife analysis of variable importance to test gain was used to assess the contribution of variables to model predictive success.

The most used SDM evaluation metric is the area under the curve (AUC) of the receiver operating characteristic (ROC) curve (Merow et al., 2013). AUC can be problematic when using presence‐only data as it must distinguish between presence and true absence (Allouche et al., 2006); though our restriction of background points across Europe, while imperfect, will have gone some way to minimizing false negatives while our models at the extent of Ireland conformed to this assumption. AUC is heavily influenced by the extent of model prediction and can be artificially inflated (Smith, 2013). Thus, the models were tested with different validation methods: AUC, sensitivity, specificity, omission rate, percentage correct, and true skill statistics (Allouche et al. 2006).

Heatmaps of the continuous probability of environmental suitability (hereafter, referred to as suitability) were binarized into gray scale maps of likely suitable conditions (hereafter, referred to as the suitable bioclimatic envelope) using the maximum test sensitivity plus specificity (MaxTSS) threshold, which optimized models using their ability to predict test rather than training data (Nameer, 2020; Smeraldo, 2020). Models were temporally extrapolated into future climatological conditions assuming low (RCP4.5) and high (RCP8.5) emission scenarios.

Suitability values per cell were compared between current and each future climate scenario using paired *t* tests. Change in the suitable bioclimatic envelope (number of suitable/unsuitable cells) was assessed between current and each future climate scenario using 2 × 2 chi‐square contingency tests. Percentage change in suitability and the number of suitable grid cells were calculated between current conditions and future conditions (Bosso, 2017; Wei et al., 2018).

### 2.6 Modeling spawning

For Ireland, egg string numbers at each pond visit across all surveys were fitted as the dependent variable in a generalized linear mixed model (GLMM), fitting Pond_ID as a random factor to account for replicate surveys per pond. Linear modeling techniques are vulnerable to model leverage due to collinearity. Climate and habitat variables are highly collinear and could not be fitted separately in the same model. Thus, sets of climate and habitat variables were each reduced in separate principal component analyses (PCA) to create orthogonal axes. Only axes with an Eigenvalue > 1 were retained for inclusion in analysis (Kaiser, 1960). Climate PCA axis scores at the point of Survey*_t_* and seasonal effects covering Spring*_t_* as well as temporally lagged effects covering Spring*_t_*_−1_, Summer*_t_*_−1_, Autumn*_t_*_−1_, and Winter*_t_*_−1_ (those of the preceding year) were fitted. Habitat PCA axis scores were also fitted. Projections were made using future climatic conditions under low (RCP 4.5) and high (RCP 8.5) emission scenarios for each spatially explicit survey location. For prediction, future seasonal averages were used for both current (*_t_*) and lagged effects (*_t_*_−1_). A similar GLMM was constructed for the first spawning date for each pond each year fitting Julian day as the dependent variable and Pond_ID as random factor. In this case, only climate PCA axes for Spring*_t_* were fitted as independent variables. Predicted egg string numbers and Julian day of first spawning were compared between current and each future climate scenario using a two‐way ANOVA fitting period and emission scenario with median, interquartile ranges, and 95% confidence intervals plotted as boxplots. Percentage change in predicted egg string numbers was calculated between current and future conditions. At the aggregate level of each survey year, the total number of potential breeding ponds that formed each spring and the cumulative total number of egg strings deposited were related to rainfall during Winter*_t_*_−1_ using Spearman's correlation. All analyses were performed using IBM SPSS Statistics v26.

## 3 RESULTS

### 3.1 Niche characterization

**FIGURE 2:**
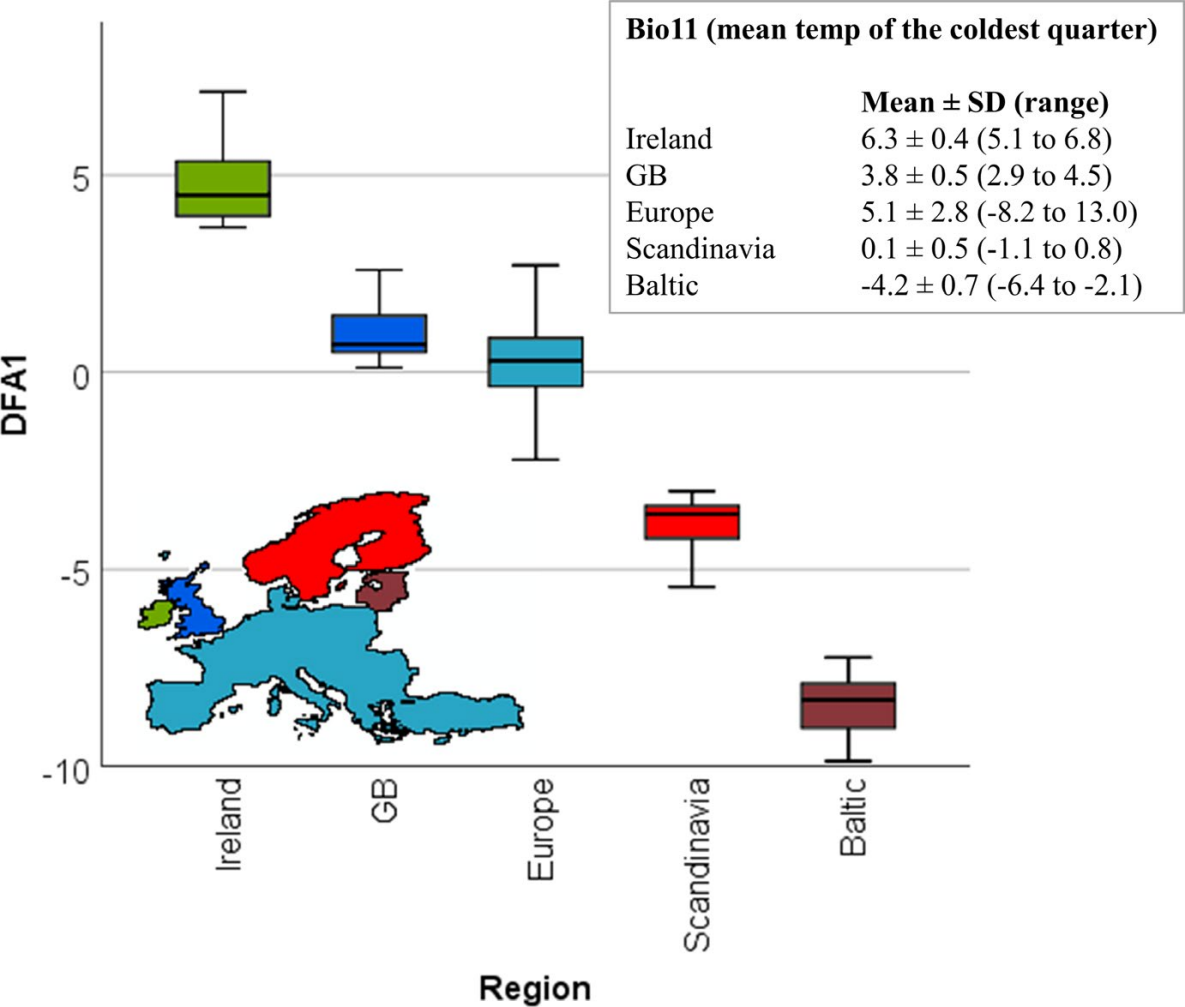
Bioclimatic‐habitat DFA1 axis scores for Natterjack toad records for each European region with the insert showing mean ± *SD* in bio11 (mean temperature of the coldest quarter) which was the main contributing variable to DFA1

Discriminant function analysis (DFA) captured the bioclimatic‐habitat variation of the Natterjack toad's niche space throughout its European range. DFA1 captured 85% of variation and is the only axis with an eigenvalue > 1 (Table 1). DFA1 scores differed significantly between regions (*F_df_*_=4,6,154_ = 2,810, *p* < 0.001) with all pairwise comparison tests being significant (*p* < 0.001), exhibiting a very strong trend from Ireland in the west to the Baltic in the east (Figure 2). DFA1 was characterized predominantly by mean temperature of the coldest quarter (bio11), that is, average winter temperatures which ranged from 6.3 ± 0.4°C in Ireland to −4.2 ± 0.7°C in the Baltic (Figure 2 insert).

**TABLE 1:** Discriminant function analysis (DFA) of WorldClim bioclimatic and CORINE habitat variables for Natterjack toad species records throughout Europe

	DFA1	DFA2	DFA3	DFA4
Eigenvalue	1.827	0.186	0.104	0.033
% of Variance	85	9	5	2
Cumulative %	85	94	98	100
**Variable**	**Loadings**			
bio11	**0.380** ^a^	0.168	−0.213	0.114
bio2	0.196	**0.652** ^a^	−0.296	0.106
bio10	0.149	**0.434** ^a^	−0.374	0.173
dist_to_coast	0.039	**0.418** ^a^	−0.076	0.063
sparse	−0.043	**−0.345** ^a^	−0.174	0.343
bio1	0.273	**0.341** ^a^	−0.293	0.120
scrub	0.005	**0.238** ^a^	−0.139	0.115
grassland	0.017	**−0.217** ^a^	0.163	−0.047
bio16	0.048	−0.098	**0.703** ^a^	0.379
bio12	0.058	−0.156	**0.624** ^a^	0.302
coastal_habs	0.057	−0.262	**0.591** ^a^	−0.351
bio17	0.041	−0.163	**0.360** ^a^	0.079
freshwater	−0.060	−0.027	0.016	**−0.095** ^a^

^a^ Symbolized variables that significantly (*p* < 0.05) contributed to each DFA axis.

### 3.2 Species distribution models

SDM predictive performance varied marginally between models at different extents and using different climate data but all models had good predictive success with AUC > 0.7 (Table 2), while TSS values varied between 0.524 ± 0.006 (Europe_WorldClim_) and 0.800 ± 0.184 (Ireland_ICHEC_) (Table S2). The Ireland_ICHEC_ model performed better than the Ireland_WorldClim_ model which, in turn, was better than the Europe_WorldClim_ model.

**TABLE 2:** Species distribution model average sample sizes (background, training, and test) per run (×4 replicate runs) and average model evaluation metrics ± *SE*

Description	Parameter	SDM		
		Europe_WorldClim_	Ireland_WorldClim_	Ireland_ICHEC_
Sample size (*n*)	Background	14,514	7,045	5,491
	75% training	4,625	18	9
	25% test	1,541	6	3
Model evaluation_test_	AUC_nothreshold_	0.832 ± 0.006	0.967 ± 0.021	0.984 ± 0.009
	AUC_MaxTSS_	0.767 ± 0.005	0.896 ± 0.078	0.900 ± 0.092
	Sensitivity_MaxTSS_	0.883 ± 0.018	0.875 ± 0.144	0.833 ± 0.192
	Specificity_MaxTSS_	0.651 ± 0.023	0.917 ± 0.047	0.966 ± 0.017
	Omission rate_MaxTSS_	0.117 ± 0.018	0.125 ± 0.144	0.167 ± 0.192
	Proportion correct_MaxTSS_	0.682 ± 0.018	0.917 ± 0.047	0.966 ± 0.017

Note

Subscript MaxTSS = maximum test sensitivity plus specificity, used as a classification threshold for suitable/unsuitable bioclimatic conditions.

**FIGURE 3:**
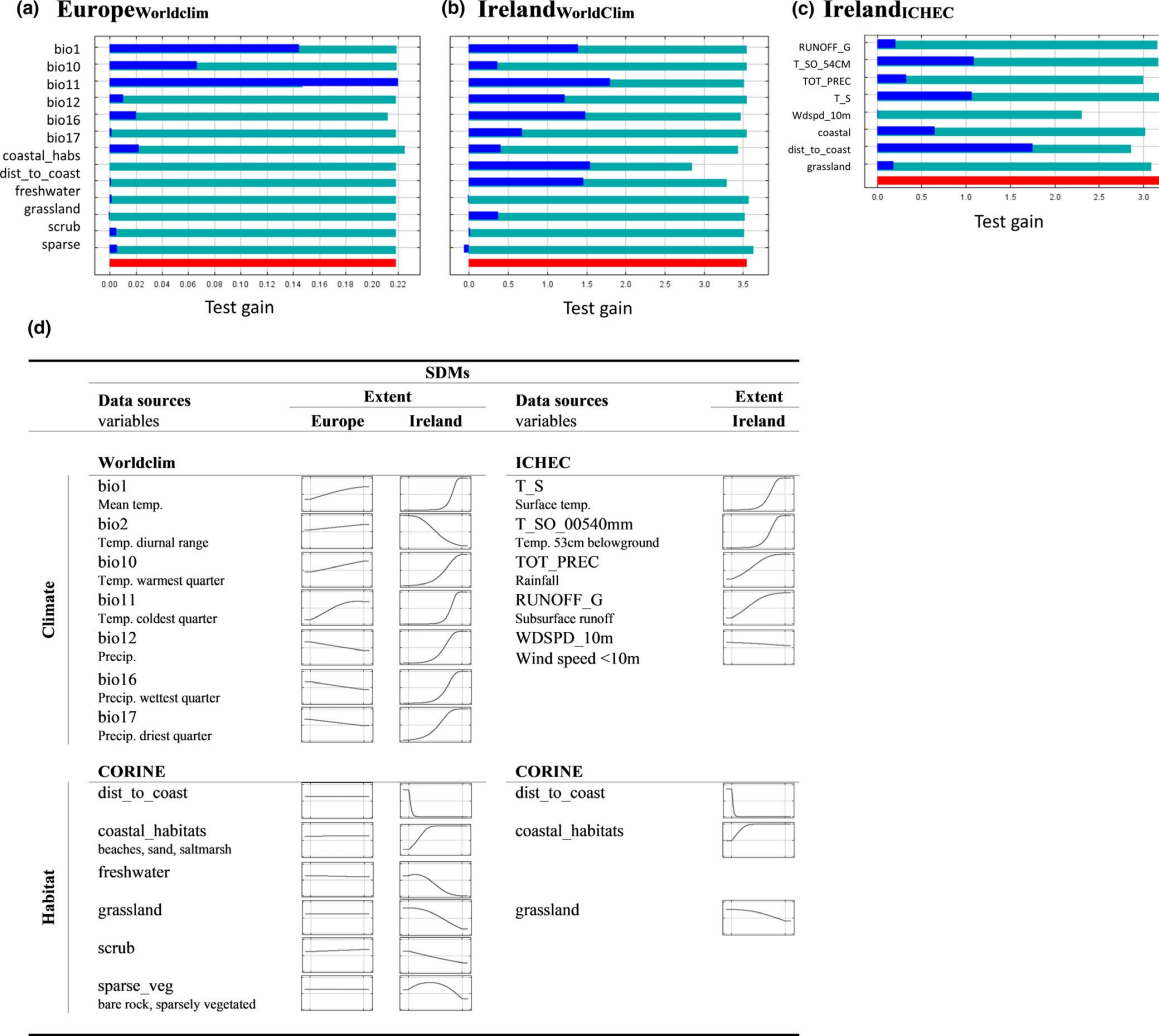
Jackknife test of variable importance to test gain for SDMs built at the extent of (a) Europe using WorldClim, (b) Ireland using WorldClim, and (c) Ireland using ICHEC climate variables. Note the *x*‐axis varies between models with interpretation based on the relative size of the bars within each plot. (d**)** Species response curves showing suitability (line) for species presence (*y*‐axes vary from 0 to 1) with variation in each climate and habitat variable (*x*‐axes varying from lowest to highest values within each variable). Axes values have been removed for simplicity. Curves reflect dependence of predicted suitability both on the named variable and on the dependencies induced by correlations between the named variable and all other variables (i.e., they are adjusted for multicollinearity)

**FIGURE 4:**
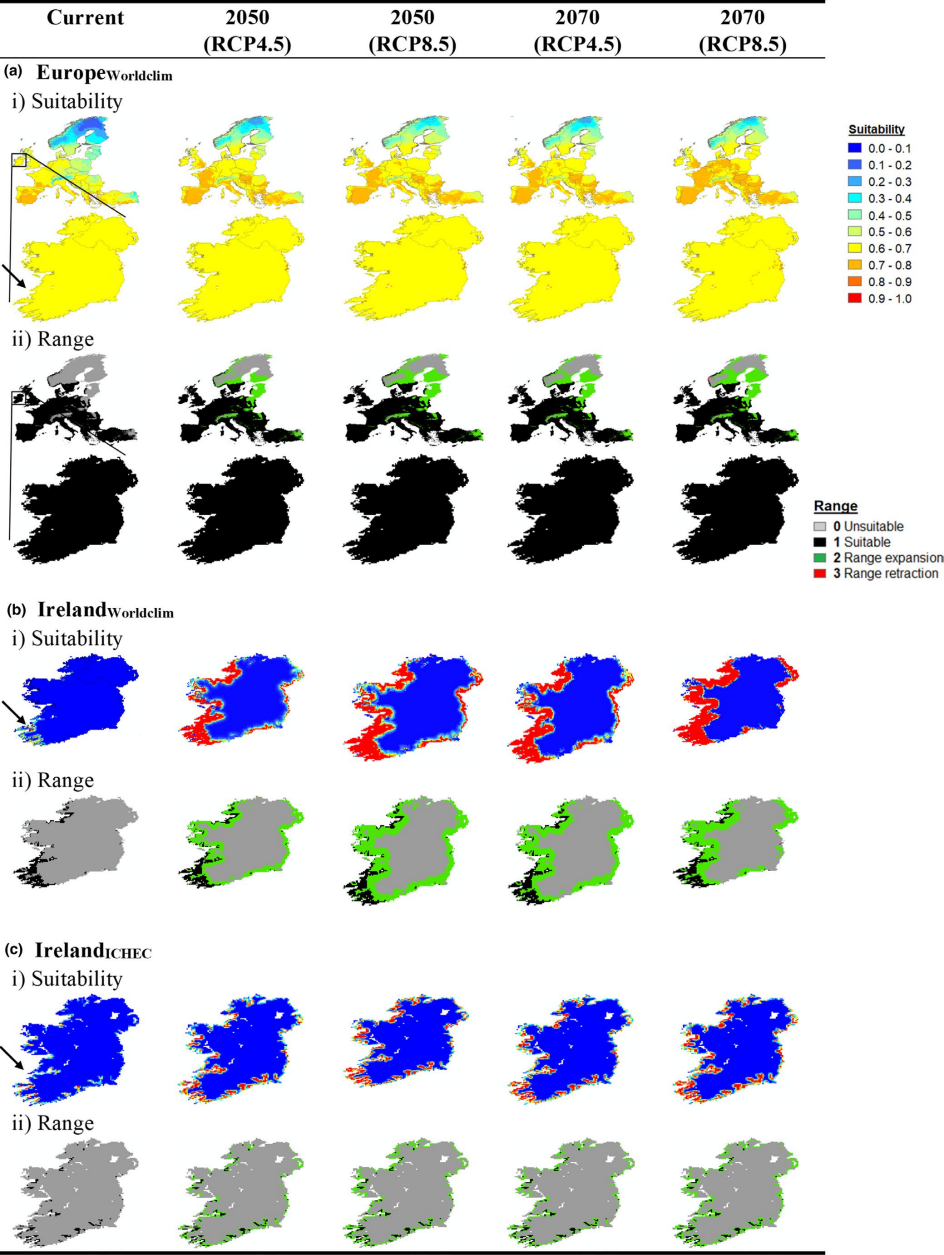
Three Natterjack toad SDMs: (a) Europe using WorldClim, (b) Ireland using WorldClim, and c) Ireland using ICHEC climate data showing (i) suitability (continuous predicted probability) and (ii) suitable range (binary suitable/unsuitable using the MaxTSS threshold) showing range expansion (green) or retraction (red) from current conditions to future climate scenarios (columns). The native range of the Natterjack toad in Ireland is indicated by the arrow

The Europe_WorldClim_ model test gain was contributed to most by bio11 (mean temperature of the coldest quarter), bio1 (mean annual temperature), and bio10 (mean temperature of the warmest quarter) with likelihood of presence greatest at higher temperatures (Figure 3). Habitat (mostly sparse vegetation and scrub) contributed little to the overall model. The Europe_WorldClim_ model (% correct = 0.682; Table 2) predicted suitable conditions for Natterjack toads throughout Europe, including all of Ireland, except for Scandinavia (Figure 4). Suitability for Natterjack toads increased significantly (*p* < 0.001) under all future climate scenarios in all European regions (Table S3) with greatest improvement (% change) in Scandinavia and the Baltic (Figure S3). Consequently, the suitable bioclimatic envelope (number of suitable grid cells) increased most significantly in both regions but also increased across mainland Europe and Great Britain (Figure 4, Table S4 and Figure S4). Despite a significant increase in suitability (Table S3), Ireland was predicted to be 100% suitable (above the MaxTSS threshold) under current conditions and to remain so regardless of the greenhouse gas emission scenario or future timeframe (Figure 4, Table S4 and Figure S3).

The Ireland_WorldClim_ model test gain was contributed to most by bio11 (mean temperature of the coldest quarter), bio16 (precipitation of the wettest quarter), and bio1 (mean annual temperature) but also coastal habitats (all positive relationships) and distance to coast (a strong negative relationship). The Ireland_WorldClim_ model (% correct = 0.917, Table 2) predicted that only the southwest of Ireland was currently suitable for the Natterjack toad with greatest suitability matching the cells that are currently occupied (Figure 4). By 2070 under the high emission scenario (RCP8.5), a > 1,000% increase in suitability (Table S3; Figure S3) and 42% increase in suitable cells (Table S4; Figure S3) were predicted with virtually the entire Irish coast (Figure 4) likely to be suitable.

Unlike the two WorldClim models, the Ireland_ICHEC_ model test gain was contributed to most, not by climate, but by a negative relationship with distance to coast followed by positive relationships with surface temperature (T_S), and soil temperature (T_SO_00540mm), coastal habitats, rainfall (TOT_PREC), subsurface run‐off (RUNOFF_G), and negative relationships with grassland and wind speed (WDSPD_10m) contributing least (Figure 3). The Ireland_ICHEC_ model (% correct = 0.966, Table 2) predicted that small patches of the Irish coast, mostly in the south and southwest, were currently suitable for the Natterjack toad, with these regions expanding throughout the 21st century (Figure 4). The suitability of Ireland was projected to increase by 41% and the number of suitable cells by 291% by the 2070s under the high emission scenario (Tables S3 and S4; Figure S3). When restricted to just those cells currently occupied by Natterjack toads, suitability increased by 27% by the 2070s under the high emission scenario (Table S3).

### 3.3 Modeling spawning

Prior to modeling fecundity and first spawning dates using GLMM, climate variables were reduced to two principal components: PC1 (eigenvalue 1.959) positively loaded for total precipitation (+0.762), subsurface run‐off (+0.817), and wind speed (+0.843), hereafter referred to simply as “rainfall” and PC2 (eigenvalue 1.900) positively loaded for surface temperature (+0.975) and soil temperature (+0.975), hereafter referred to simply as “temperature.” Habitat was captured by a single principal component: PC3 (eigenvalue 1.505) negatively loaded for grassland (−0.868) and positively loaded for coastal habitats (+0.868), hereafter referred to as a grassland‐dune gradient.

**FIGURE 5:**
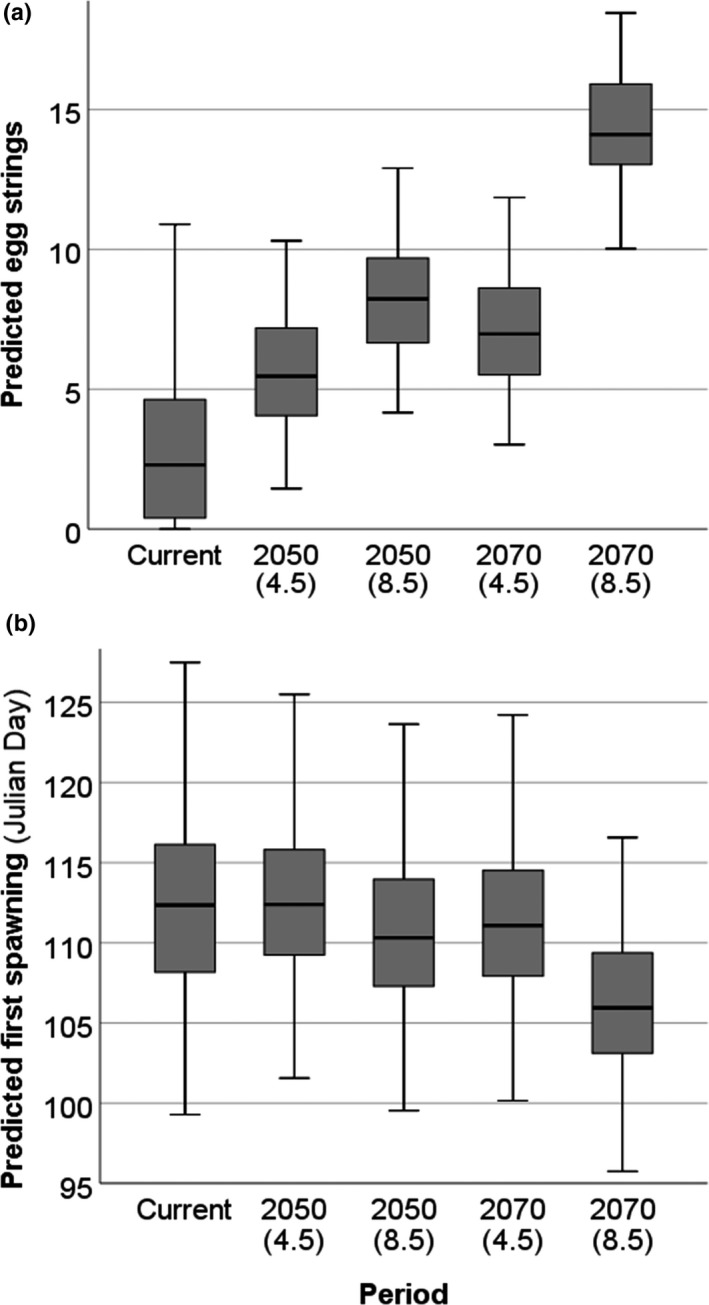
GLMM predictions of (a) fecundity and (b) first spawning dates (Julian day) between current conditions and the mid‐ to late 21st century under low (RCP4.5) and high (RCP8.5) GHG emission scenarios

Natterjack toad fecundity was negatively related to temperature during the Survey*_t_* and positively during Summer*_t_*_−1_. Fecundity was negatively related to rainfall during Summer*_t_*_−1_ but positively during Winter*_t_*_−1_ (Table 3). At the aggregate population level, the number of ponds that formed annually was significantly positively correlated with rainfall during Winter*_t_*_−1_ (*r*_s_ = 0.778, *p* = 0.023) and the cumulative total number of egg strings deposited throughout the Natterjack toad's Irish range each breeding season (*r*_s_ = 0.778, *p* = 0.023). Fecundity (weekly numbers of egg strings deposited) was predicted to increase significantly as the century progresses (*F_df_*_=1,16,946_ = 7,641.089, *p* < 0.001), being significantly higher under the high than low emission scenarios (*F_df_*_=1,16,946_ = 12,857.447, *p* < 0.001) and increasing by 104% from 2.7 egg strings per pond visit currently to 5.5 eggs strings during 2050 RCP4.5, by 201% to 8.1 eggs strings during 2050 RCP8.5, by 158% to 7.0 eggs strings during 2070 RCP4.5, and by 425% to 14.2 egg strings during 2070 RCP8.5 (Figure 5).

**TABLE 3:** GLMM results for the effects of seasonal temporal lags in climate on Natterjack toad (a) fecundity and (b) first spawning dates

Variables	Model				
	*F*	*β* ± *SE*	*n*.*df*	d.*df*	*p*
(a) Fecundity (egg strings) *F*_df=13,3,376_ = 9.783, *p* < 0.001					
Survey*_t_*					
Temperature	48.113	−2.119 ± 0.306	1	3,376	**<0.001**
Rainfall	0.498	0.207 ± 0.294	1	3,376	0.480
Spring*_t_*					
Temperature	0.543	0.694 ± 0.941	1	3,376	0.461
Rainfall	2.947	1.528 ± 0.890	1	3,376	0.086
Winter*_t_*_−1_					
Temperature	1.745	1.271 ± 0.962	1	3,376	0.187
Rainfall	18.395	3.927 ± 0.916	1	3,376	**<0.001**
Autumn*_t_*_−1_					
Temperature	0.959	−0.621 ± 0.635	1	3,376	0.327
Rainfall	2.682	−1.456 ± 0.889	1	3,376	0.102
Summer*_t_*_−1_					
Temperature	6.746	2.339 ± 0.900	1	3,376	**0.009**
Rainfall	11.996	−3.024 ± 0.873	1	3,376	**0.001**
Spring*_t_*_−1_					
Temperature	0.922	0.814 ± 0.847	1	3,376	0.337
Rainfall	0.114	−0.347 ± 1.030	1	3,376	0.736
Habitat					
Grassland‐dunes	1.087	0.478 ± 0.459	1	3,376	0.297
(b) First spawning date (Julian day) *F_df_*_=2,222_ = 3.442, *p* =0.034					
Spring*_t_*					
Temperature	6.452	−3.219 ± 1.267	1	222	**0.012**
Rainfall	0.797	−1.195 ± 1.339	1	222	0.373

Significant results are in bold.

First spawning dates, currently occurring on average by 21 or 22 April (Julian day 112), were negatively related to Spring*_t_* temperatures but unrelated to rainfall (Table 3). Initiation of spawning was predicted to occur significantly earlier as the century progresses (*F_df_*_=1,1,121_ = 58.497, *p* < 0.001) and earlier under the high than low emission scenario (*F_df_*_=1,1,121_ = 85.229, *p* < 0.001) with future predictions suggesting advancement by, on average, 6 days to 15 or 16 April (Julian day 106) by the 2070s RCP8.5 (Figure 5).

## 4 DISCUSSION

Models of the Natterjack toad environmental suitability, bioclimatic envelope, fecundity, and phenology suggest the species is highly responsive to climate but inhabits a wide range of climatic and habitat conditions throughout Europe. Our results suggest projected climate change may make Europe, including Ireland, more suitable for the species. Fecundity in Ireland is projected to increase with earlier spawning due to increasingly favorable conditions as the 21st century progresses, especially under a high greenhouse gas emission scenario.

### 4.1 Niche characterization

Discriminant function analysis suggested that mean temperature of the coldest quarter (winter temperatures) is most limiting to the range of the Natterjack toad with most of Scandinavia, the Baltic, and Eastern Europe currently unsuitable, matching its known range edge margins. In the Northern Hemisphere, climate change is predicted to lead to milder winter temperatures and a decrease in duration of cold periods and snow cover (IPCC, 2013; Räisänen et al., 2004). In alpine and boreal habitats, milder winter temperatures have been shown to promote amphibian population viability (McCaffery & Maxell, 2010; Üveges et al., 2016). Thus, reduced winter severity in the future could be potentially beneficial for the Natterjack toad, most notably in Scandinavia and the Baltic, where the species exists at the extremes of its thermal limits. More generally, the suitability of central Europe for the species may also increase with climate change as conditions more characteristic of its current core range in Iberia and France become more widespread.

### 4.2 Species distribution models

The SDM at the extent of Europe failed to predict the species' restricted range in Ireland and Great Britain, and its more widespread range in the Baltic. This is likely due to the extreme range of conditions tolerated by the Natterjack toad at its range edge margins with the model failing to account for colonization history and local adaptation. Populations have adapted to breed in water with salinity above the species lethal threshold (Gomez‐Mestre & Tejedo, 2003), in the presence of other amphibian competitors (Gomez‐Mestre & Tejedo, 2002), or in large lakes (Reyne et al., 2020), suggesting a high degree of plasticity and adaptation to less favorable conditions. The impact of climate change can be highly variable among populations with some being more resilient to climatic variation (Griffiths et al., 2010; Muths et al., 2017), indicating highly context‐dependent responses. Our SDMs at the extent of Ireland, while failing to capture broader environmental tolerances of the species throughout Europe, accurately reflected the Natterjack toad's highly restricted range in the southwest of Ireland. Both WorldClim and ICHEC climate data indicated that suitable bioclimatic‐habitat conditions currently exist more widely in the southwest of Ireland but also around the Irish coast than are currently occupied. SDMs fail to account for the chronology of biogeographical events after the last glaciation leading to Ireland's colonization by the Natterjack toad (Rowe et al., 2006) and human impacts through ancient landscape modification and modern habitat destruction and deterioration. Natterjack toads, like most amphibians, have limited dispersal capabilities (Smith & Green, 2005), suggesting that once historically restricted to the southwest of Ireland they were unable to subsequently disperse to other suitable regions regardless of their proximity.

A positive impact of climate change on amphibian populations has been projected for other European species (Carey & Alexander, 2003; Corn, 2005; Dolgener et al., 2014). Araújo et al. (2006) predicted that 42 amphibian species in Europe will expand their distribution northward by 2050. However, the simple existence of suitable conditions does not necessarily mean that species ranges will expand as expansion will depend on each species' dispersal ability, the existence of vital source populations, suitable habitats, and pathways for dispersal (Girardello et al., 2010). When comparing the distribution of Iberian amphibian species between two time periods (1901–1990 vs. 2000–2015), almost no shifts in distribution were observed, despite changes in climatic conditions (Enriquez‐Urzelai et al., 2019). Under stressful conditions, such as hot and dry weather, amphibians tend to seek refuge and travel shorter distances, thus further restricting their already limited dispersal capabilities (Chan‐McLeod, 2003; Roe & Grayson, 2008). Considering amphibian dispersal limitations, species will likely fail to track shifts in their suitable bioclimatic envelope in the future (Lawler et al., 2010). It is important to note that in the current study, species occurrence data represented a snapshot of distribution with toads more likely to be recorded during the breeding season when they and their spawn were more conspicuous. Variation in habitat requirements for breeding, foraging, and winter refugia exists (Denton & Beebee, 1993), which are not considered in the current analysis. Thus, spatially explicit predictions of models should be treated with caution with models being indicative of the likely trajectory of the impact of climate change only.

### 4.3 Modeling spawning

Predictions of fecundity and first spawning dates in Ireland supported SDM predictions of increasing suitability with numbers of eggs strings predicted to increase and initiation of spawning likely to occur earlier in the future, especially under a high greenhouse gas emission scenario. The number of egg strings deposited was most strongly associated with lower temperatures during the breeding season (April to July) as most are deposited in late spring (April to May) when its cooler than during early summer (June to July) when it is warmer. Thus, spring temperature may be more informative by its effect on the initiation of spawning rather than fecundity per se. Spawning was earlier when spring surface and soil temperatures were warm, which may be linked to earlier emergence from hibernacula, and later when they were cool. Amphibians exhibit the greatest phenological response of any taxa to climate change (Parmesan, 2007). Shifts in reproductive timing have already been observed among various pond breeding amphibians in North America and Europe (e.g., Gibbs & Breisch, 2001; Scott et al., 2008; Todd et al., 2011; Tryjanowski et al., 2003). Early breeding can have positive effects like longer development times for tadpoles, more time to accumulate energy reserves and for development of ovaries of recently sexually matured females, enabling spawning in the next breeding season (Jørgensen, 1986; Morbey & Ydenberg, 2001; Tryjanowski et al., 2003), thus increasing fecundity, recruitment, and survival. However, advancement of the first spawning date can expose eggs and tadpoles to more variable and unpredictable weather, like freezing, or to interspecific competition. For instance, early Natterjack toad breeding can increase the niche overlap with tadpoles of early breeders like the common frog (*Rana temporaria*), thus inducing competition and potential predation (Beebee, 2002; Richter‐Boix et al., 2006). Hence, consequences of breeding earlier on population trends are hard to predict.

Natterjack toad fecundity in Ireland was positively associated with rainfall during the preceding winter, which formed more breeding pools and typically extends pond hydroperiods, especially in sand dune slacks (Reyne et al., 2019). Fecundity was also influenced by conditions of the summer in the preceding year, suggesting carryover effects may be important. Fecundity was increased when the previous summer was warm and dry, presumably benefiting invertebrate prey activity, adult toad activity and body condition (toad fecundity is positively correlated with body size; Reading, 1986), and toadlet survival, growth, and population recruitment.

### 4.4 Caveats

The interactions between climate change, range, population, and life histories are complex. Many key factors relevant to amphibian biology have not, or cannot, be parameterized and predicted for the future. While habitat was explicitly included to increase the predictive power of our models, no projections of likely future land cover change are available given the unpredictable nature of coastal habitat development, urbanization, and food production that drives agricultural change. The Natterjack toad in Ireland is regionally Red‐Listed as Endangered due to recent range contraction and population decline driven by habitat loss and deterioration (King et al., 2011). Regardless of whether climate may become more benign for Natterjack toads in Ireland, if historical and current threats and pressures continue it seems unlikely that climate will be able to mitigate ongoing declines. Climate change can lead to increased use of pesticides (Kattwinkel et al., 2011) and enhanced toxicity of environmental contaminants (Noyes et al., 2009). Declines in global invertebrates (Conrad et al., 2006; Potts et al., 2010; Winfree et al., 2009) of up to 82% have been recorded in recent decades in some regions of Europe (Hallmann et al., 2017). Changes in prey availability in addition to increased metabolic rate and calorific requirements because of warmer temperatures can decrease body condition, impacting fecundity, and recruitment (Martin et al., 2010). In Ireland, the Natterjack toad is found exclusively in coastal habitats. Climate change will cause sea‐level rise and more frequent and intense storms (IPCC, 2014), which may result in saltwater inundation of freshwater breeding ponds or sand dune erosion. Already, reduction in amphibian abundance and diversity has been observed in the USA in areas severely damaged by hurricanes (Schriever et al., 2009). Amphibians are highly vulnerable to pathogens and climate change can alter their spread and epidemiology. Kiesecker and Skelly (2001) showed that reduction in water depth leads to concentration of amphibian larvae and trematode‐infected snails, leading to significantly increased parasitism of host amphibians. Several hypotheses link climate change to increased chytrid fungus (*Batrachochytrium dendrobatidis*) infection rates, which may be a key factor in global amphibian declines (Lampo et al., 2006; Pounds & Crump, 1994; Pounds et al., 1999; Rohr & Raffel, 2010; Rohr et al., 2008). Modeling of climate change allows us to estimate its potential impact on some aspect of species biology including environmental suitability, suitable niche space, and reproduction, but without an ability to parameterize or predict other vital aspects of the environment or species adaptation capacity, such predictions may be of limited utility.

### 4.5 Conclusion

This study suggests that the Natterjack toad is a highly adaptable species, inhabiting a wide range of conditions throughout its European range, limited principally by winter temperatures. By the end of the 21st century, conditions in Europe may become more favorable for the species, most notably in Scandinavia and the Baltic but also Ireland. Should threats and pressures largely associated with declines in extent and habitat quality be resolved, we might expect that fecundity (and by extension recruitment and population size) may increase with favorable changes in phenology allowing earlier spawning and longer maturation periods. Currently, species conservation strategies in Ireland include the National Parks & Wildlife Service (NPWS) “Pond Creation Scheme” and “Head Start Programme” aimed at creating artificial farmland ponds as potential new breeding sites, while assisting colonization (and existing population augmentation) by captive rearing of toadlets for release back into the wild. Our results suggest that, given the wider distribution of suitable conditions outside their current highly restricted range in Ireland, Natterjack toads could be reintroduced to nearby areas where they previously existed and have since been extirpated. In addition, assisted migration could be employed to enable the species to colonize sand dunes and coastal grasslands beyond its historically known range, where suitable climatic conditions occur, forming the basis of a proactive and preemptive climate change adaptation strategy. However, careful evaluation of potential (re)introduction sites is required. For instance, our models predict two sand dune systems (Rossbeigh and Banna Strand) in Co. Kerry have high climatic‐habitat suitability, but field surveys report high salinity at ponds in the dune slacks at these sites (Reyne et al., 2020) making them unsuitable for breeding. Any conservation (re)introductions should be carefully planned and in accordance with IUCN Species Survival Commission Guidelines (IUCN/SSC, 2014). Moreover, as climate change progresses there is a need to reassess its impact on specific populations and adapt conservation practices accordingly.

## CONFLICT OF INTEREST

No conflict of interest to declare.

## AUTHOR CONTRIBUTIONS

**Marina Reyne:** Conceptualization (lead); data curation (lead); project administration (lead); writing – original draft (lead); writing – review and editing (lead). **Natasha E. McGowan:** Formal analysis (equal); methodology (supporting); visualization (supporting). **Jason Flanagan:** Data curation (supporting); formal analysis (equal); methodology (equal); software (equal); writing – review and editing (supporting). **Paul Nolan:** Data curation (supporting); formal analysis (equal); methodology (equal); software (equal); writing – review and editing (supporting). **Aurélie Aubry:** Funding acquisition (supporting); project administration (supporting); supervision (supporting); writing – review and editing (supporting). **Mark Emmerson:** Funding acquisition (equal); project administration (supporting); supervision (supporting); writing – review and editing (supporting). **Ferdia Marnell:** Conceptualization (equal); funding acquisition (equal); project administration (equal); resources (equal); writing – review and editing (equal). **Neil Reid:** Conceptualization (equal); formal analysis (lead); funding acquisition (lead); methodology (equal); project administration (equal); software (equal); supervision (lead); writing – review and editing (lead).

## Supporting information

Supplementary Material

## Data Availability

Reyne et al. (2021), Climate change and Natterjack toads, Dryad, Dataset, https://doi.org/10.5061/dryad.9zw3r22d0.
