# Supplementary material for: Will predicted positive effects of climate change be enough to reverse declines of the regionally Endangered Natterjack toad in Ireland?
Source: Ecol Evol. 2021 Mar 11;11(10):5049–64. doi: 10.1002/ece3.7362 (PMC8131806; doi:10.1002/ece3.7362)
Supplement: Supplementary file 1 — Supplementary Material [file ECE3-11-5049-s001.docx]

**Online Supplementary Information**

Will predicted positive effects of climate change be enough to reverse declines of the regionally Endangered Natterjack toad in Ireland?

**Running title:** *Climate change and Natterjack toads*

|  | **Variable code** | **Description** |
| --- | --- | --- |
|  |  |  |
| **a)** | **Worldclim bioclimatic variables – Europe-wide extent (2.5^o^ ~ 4km grid cells)** | |
|  | bio1 | Mean annual temperature (^o^C) |
|  | bio2 | Diurnal temperature range (^o^C) |
|  | bio10 | Mean temperature of warmest quarter (^o^C) |
|  | bio11 | Mean temperature of coldest quarter (^o^C) |
|  | bio12 | Annual total precipitation (mm) |
|  | bio16 | Precipitation of wettest quarter (mm) |
|  | bio17 | Precipitation of driest quarter (mm) |
|  |  |  |
| **b)** | **COSMO-CLM5 ensemble variables (ICHEC) – Ireland only (4km grid cells)** | |
|  | T_S | Surface temperature at ground level (^o^C) |
|  | T_SO_00540mm | Soil temperature at 54cm belowground (^o^C) |
|  | TOT_PREC | Precipitation (kg/m^2^) |
|  | RUNOFF_G | Subsurface runoff (kg/m^2^) |
|  | WDSPD_10m | Wind speed at 10m (m/s) |
|  |  |  |
| **c)** | **CORINE2018 (EEA) – Europe (2.5^o^ ~5km grid cells) and Ireland (4km grid cells)** | |
|  | dist_to_coast | Nearest perpendicular distance to the coast (km) |
|  | coastal_habs | Beaches, dunes, sand (331), salt marshes (421) and intertidal flats (423) |
|  | freshwater | Water courses (511) and Water bodies (512) |
|  | grassland | Pastures (231) and Natural grasslands (321) |
|  | scrub | Transitional woodland-shrub (324) and Fruit trees and berry plantations (222) |
|  | sparse_veg | Bare rocks (332) and Sparsely vegetated areas (333) |

**Table S1** Climatic and habitat variables used as explanatory environmental variables.

0 1,000 2,000 Kilometers

| **WorldClim** | **bio1** Mean annual temp  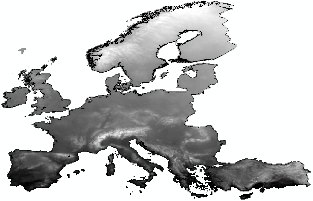 | **bio2** Diurnal temp range  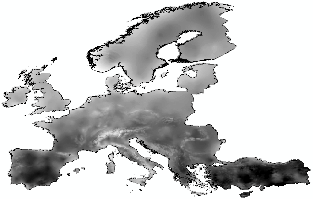 | **bio10** Mean temp of warmest quarter  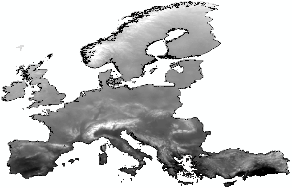 | **bio11** Mean temp of coldest quarter  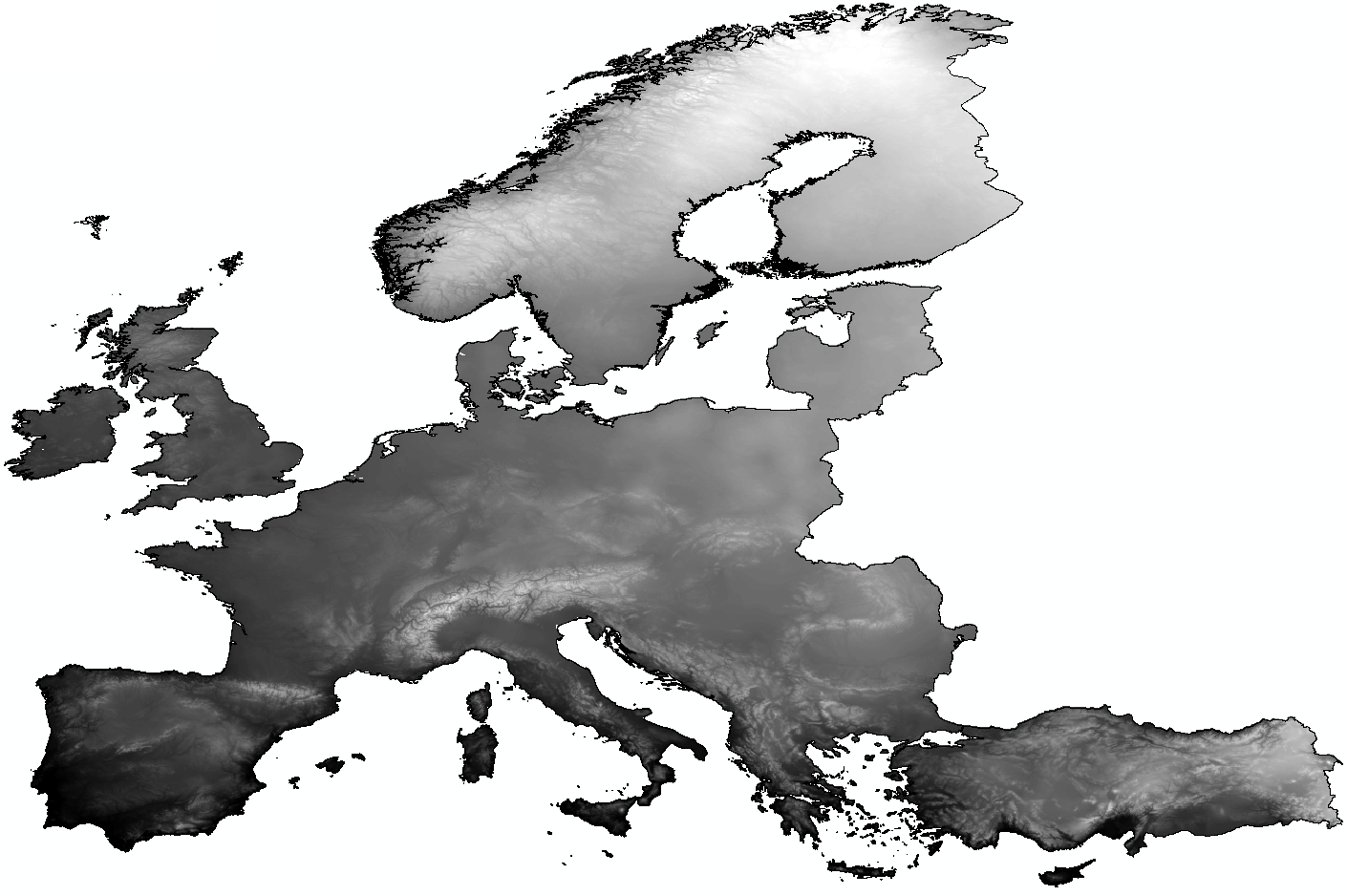 |
| --- | --- | --- | --- | --- |
|  | **bio12** Annual precipitation  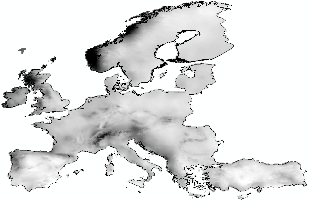 | **bio16** Precipitation of wettest quarter  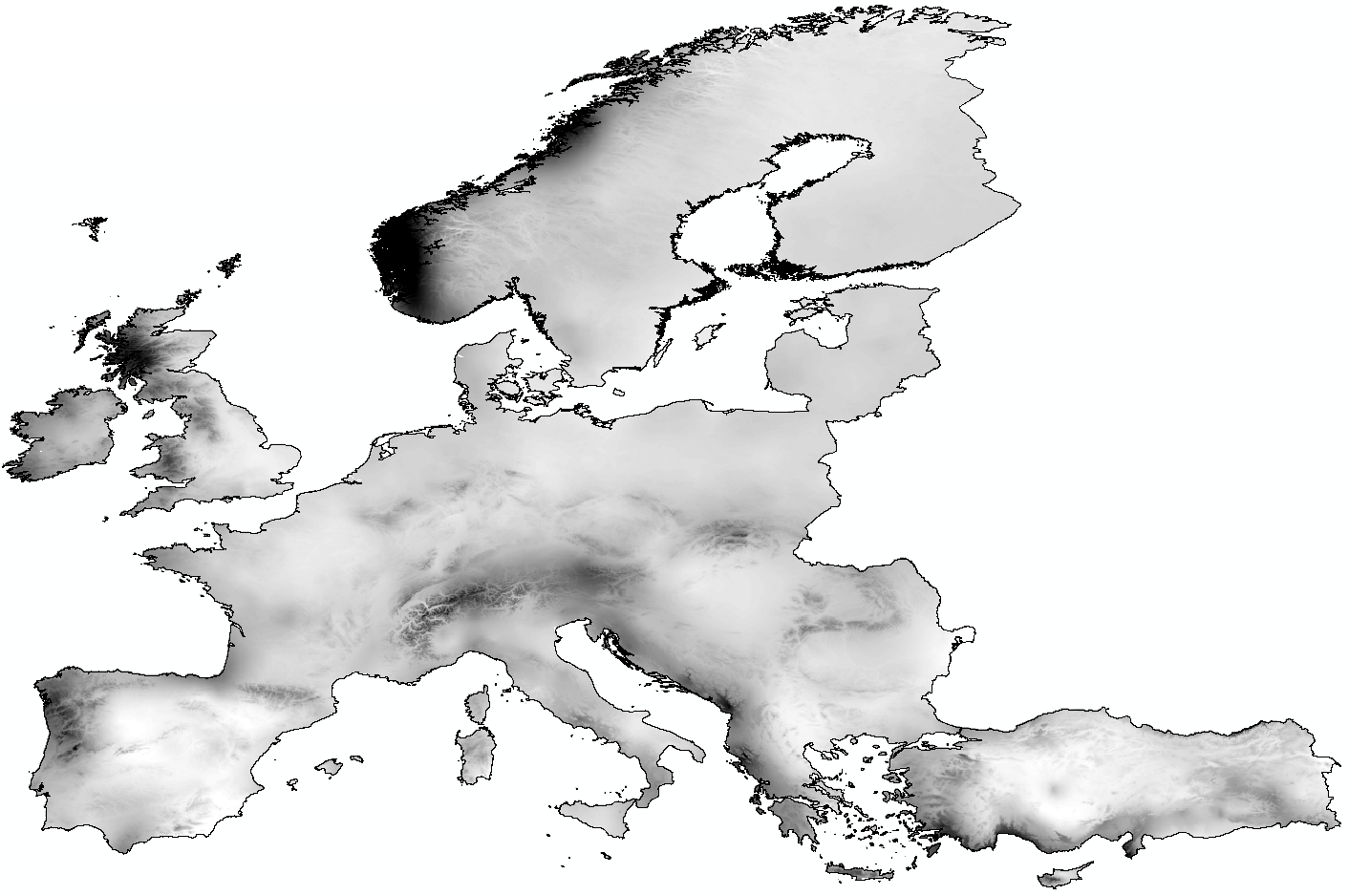 | **bio17** Precipitation of driest quarter  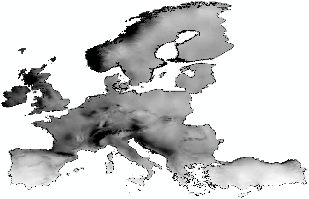 |  |
|  |  |  |  |  |
| **Habitat** | **coastal habs**  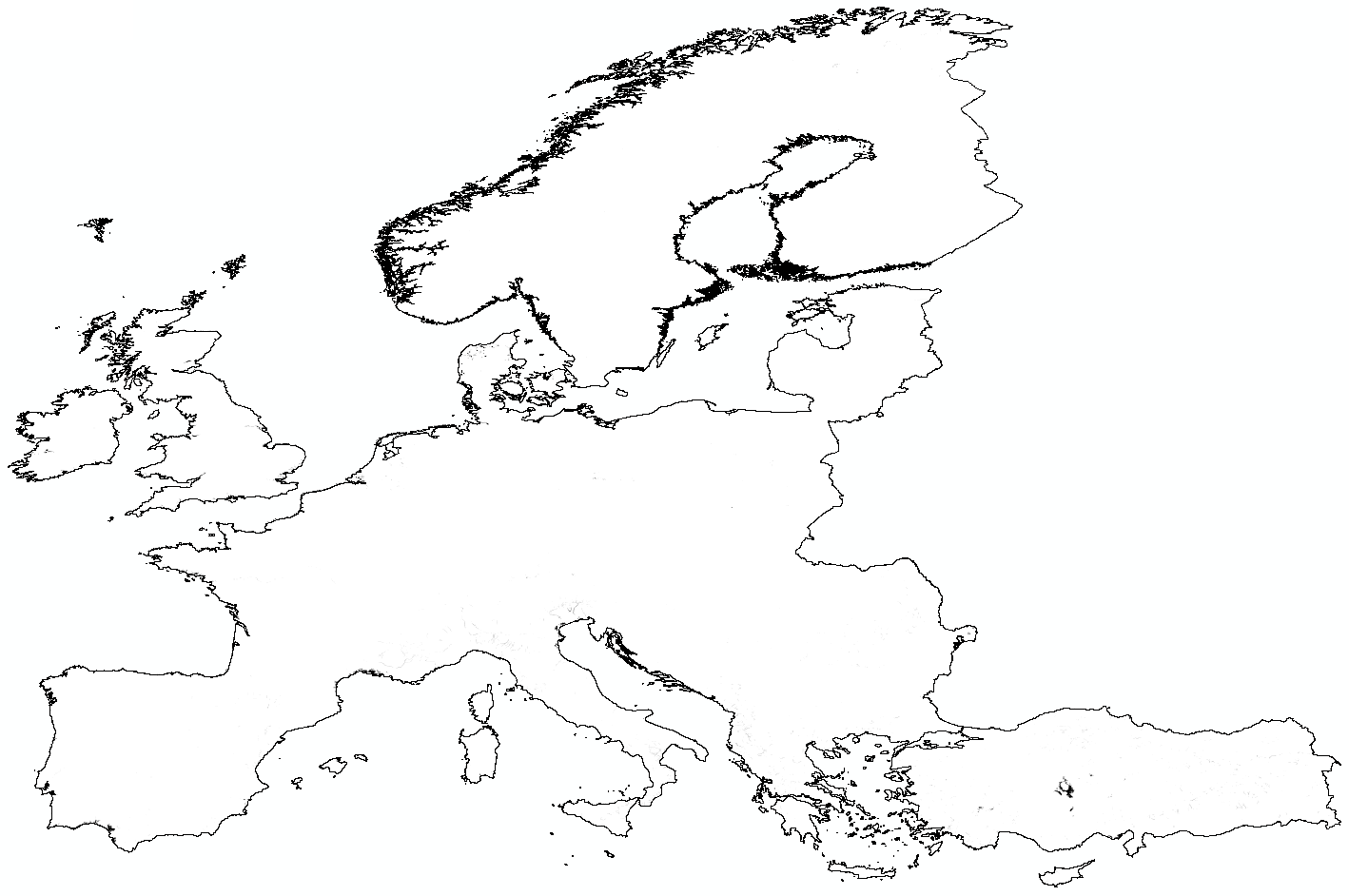 | **dist_to_coast**  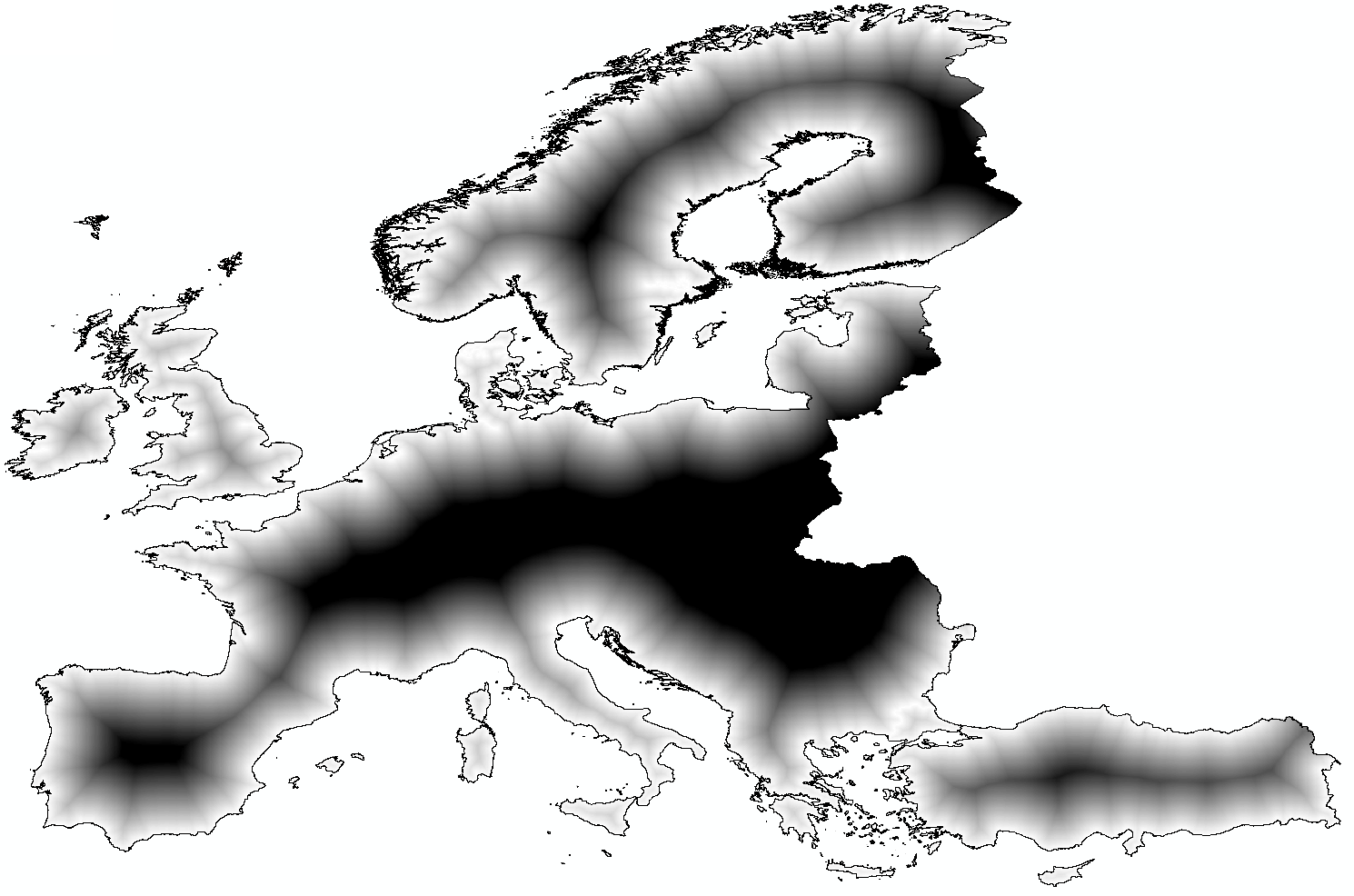 | **freshwater**  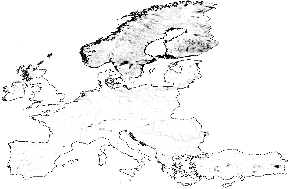 | **grassland**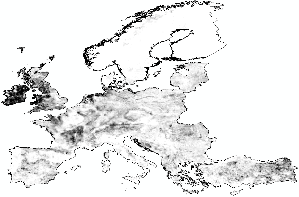 |
|  | **scrub**  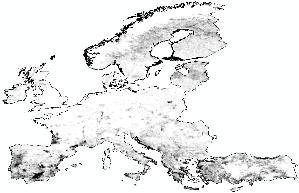 | **sparse_veg**  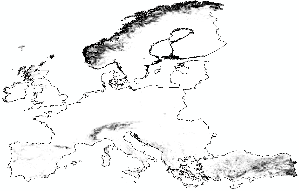 | 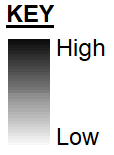 |  |

**Figure S1** Spatial variation of selected Worldclim climate variables for the current period (averaged from 1970-2000) and CORINE2018 habitat variables (using the classification in Table H.1) at a 2.5^o^ ~4km grid cell resolution throughout Europe. The same climate variables were available projected for the future periods of the 2050s and 2070s under both RCP4.5 and RCP8.5 emissions scenarios (not shown).

| **ICHEC** | **TOT_PREC**  Total precipitation 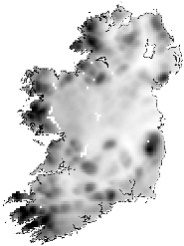 | **RUNOFF_G**  Subsurface runoff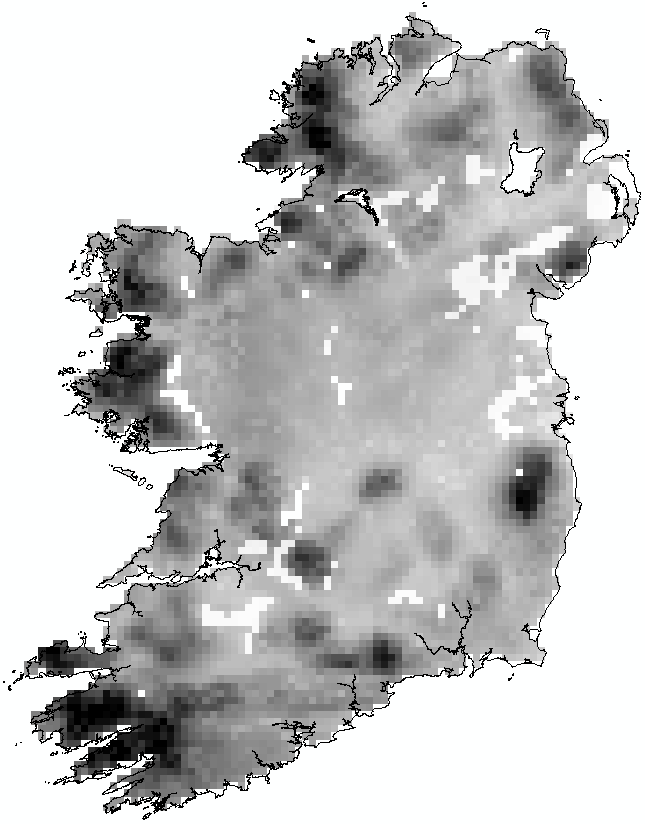 | **WDSPD_10m**  Wind speed <10m 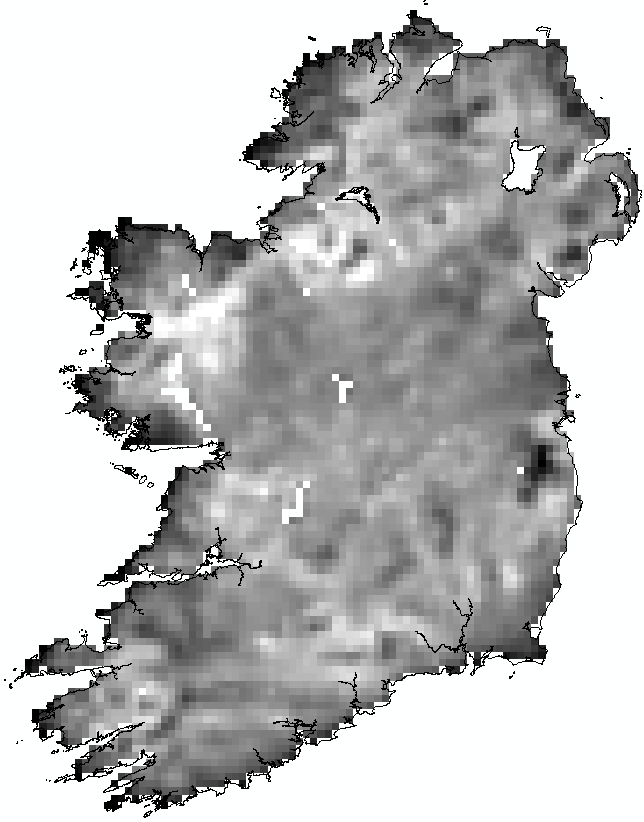 |  |  |  |
| --- | --- | --- | --- | --- | --- | --- |
|  | **T_S**  Surface temperature  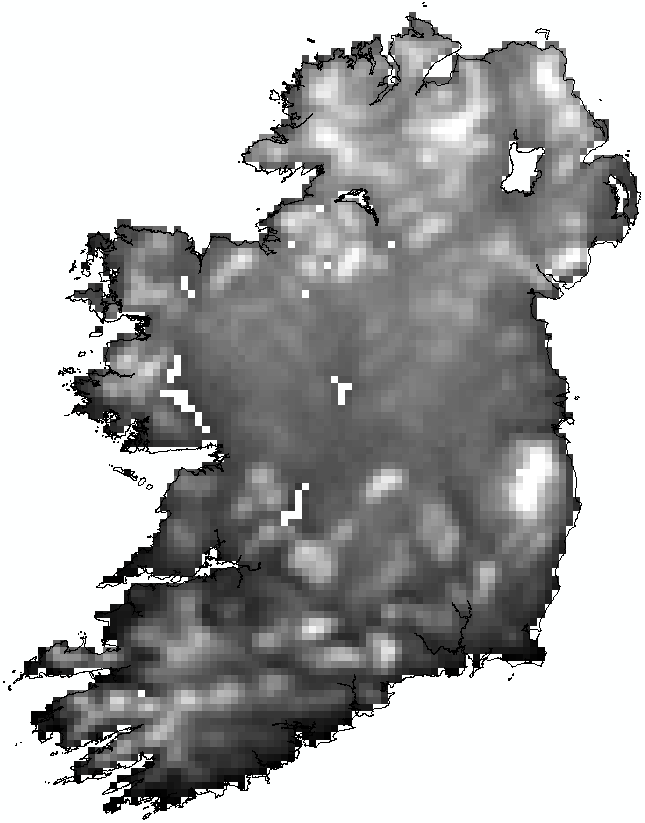 | **T_SO_00540mm**  Soil temperature  54cm below ground  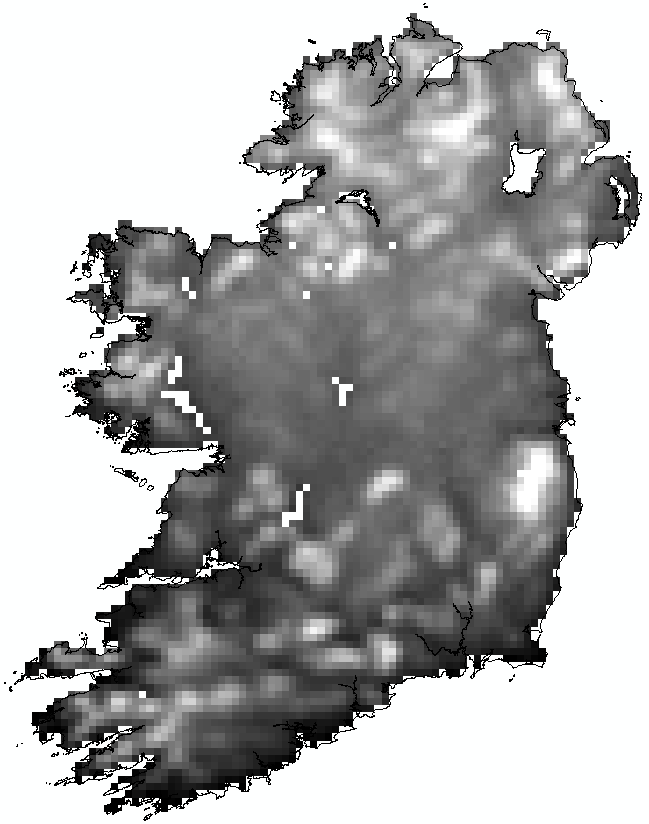 | 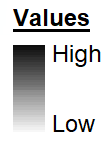 |  |  |  |
|  |  |  |  |  |  |  |
| **CORNE** | **dist_to_coast**  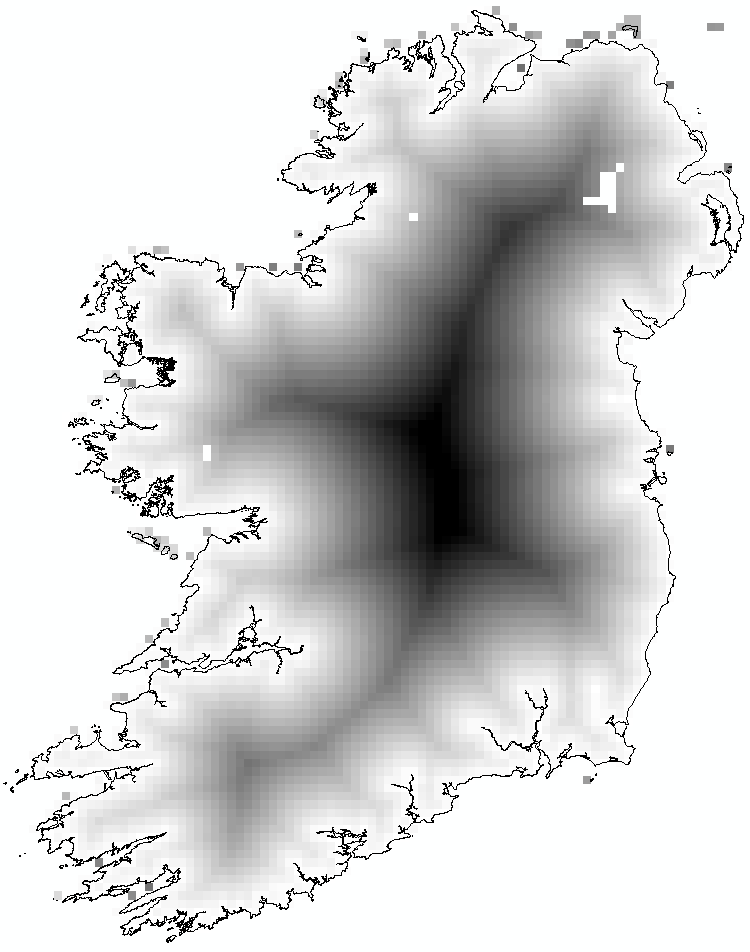 | **coastal_habs**  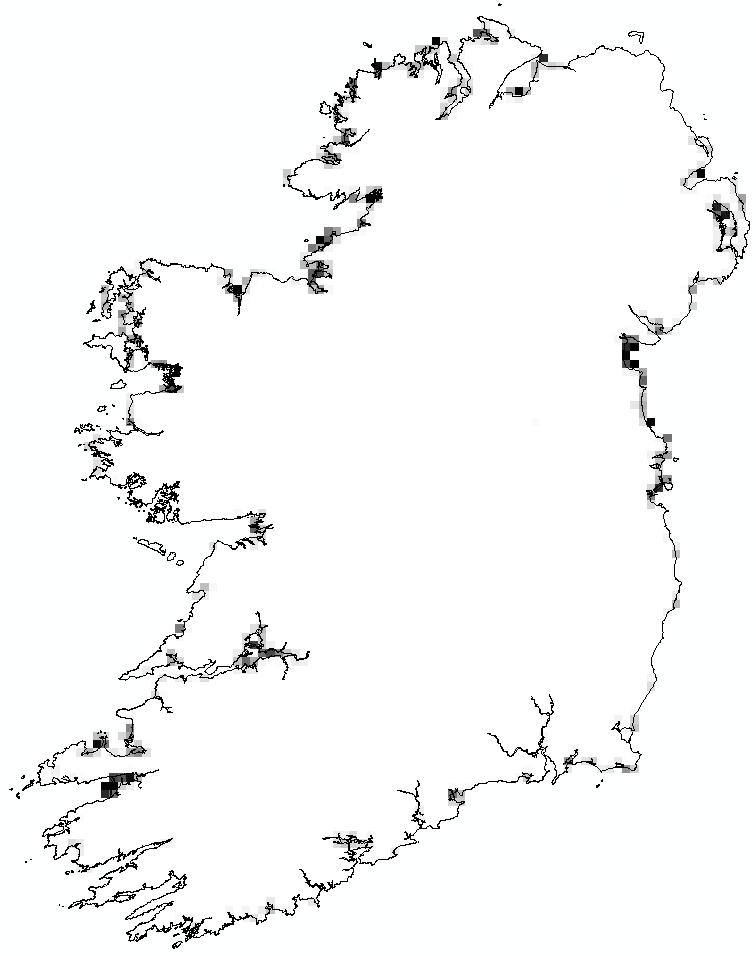 | **freshwater**  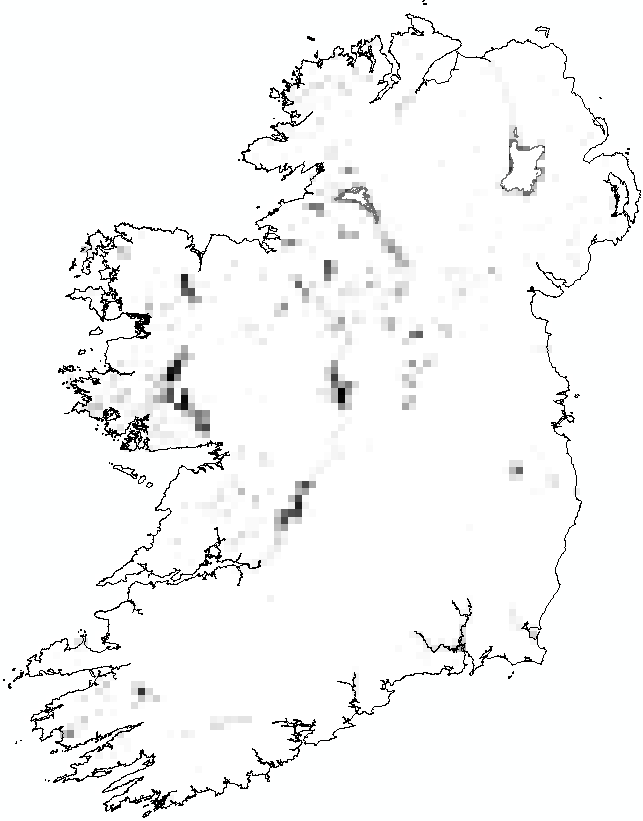 | **grassland**  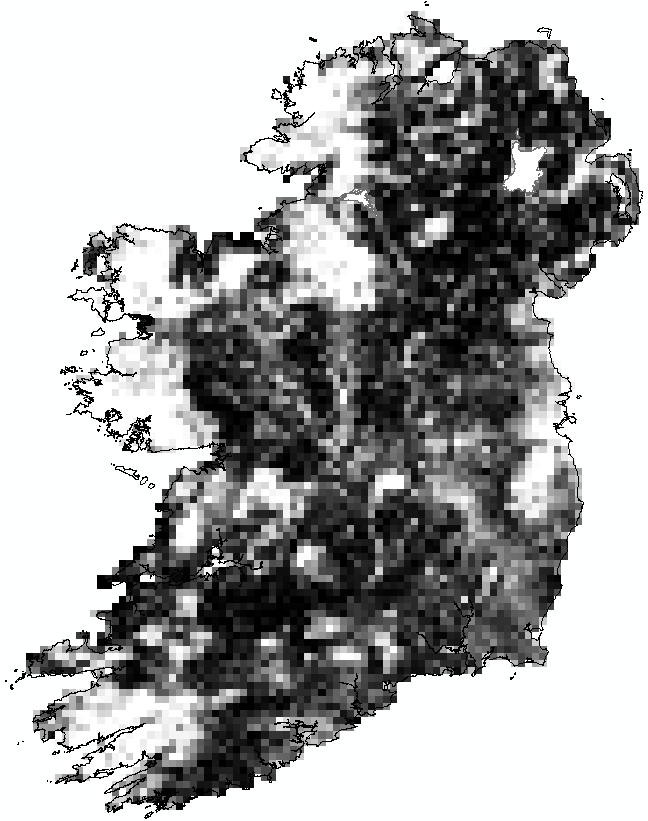 | **scrub**  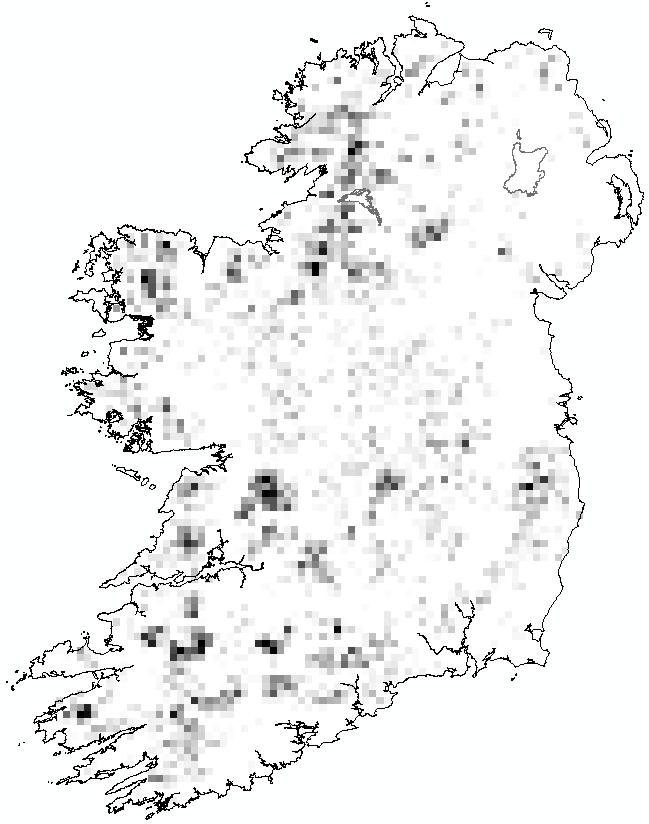 | **sparse_veg**  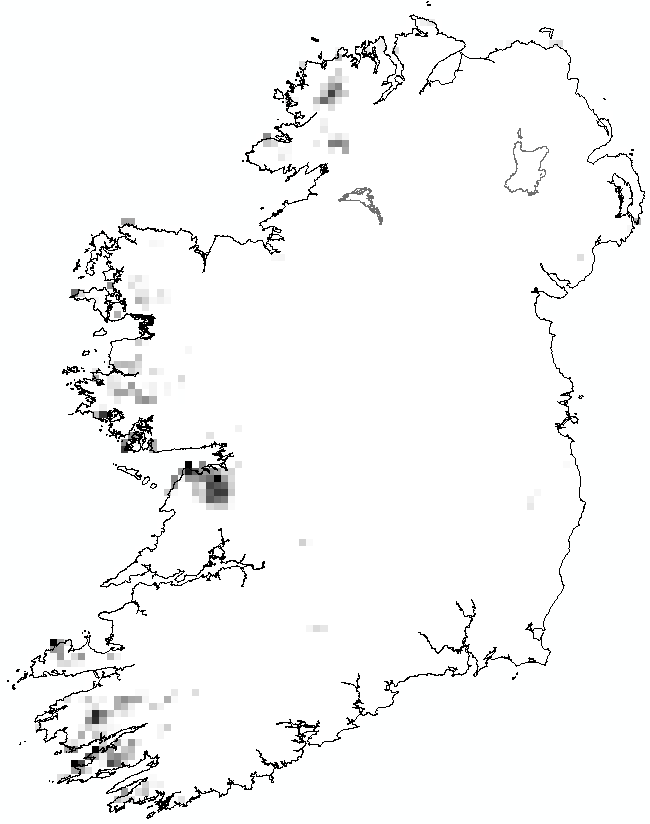 |
|  |  |  |  |  |  | 0 150 300 Kilometers |
|  |  |  |  |  |  |  |

**Figure S2** Spatial variation of selected ICHEC climate variables (derived from the COSMO-CLM5 ensemble) for the current period (averaged from 1976-2005) and selected CORINE2018 habitat variables (using the classification in Table S1) at a 4km grid cell resolution throughout Ireland. The same climate variables were available projected for the future periods: 2050s and 2080s under both RCP4.5 and RCP8.5 emissions scenarios (not shown).

**Table S2** Validation of Natterjack toad Species Distribution Models for Europe and Ireland.

|  |  | **Europe Worldclim** | **Ireland ICHEC** | **Ireland Worldclim** |
| --- | --- | --- | --- | --- |
| No threshold | AUC | 0.832 ± 0.006 | 0.984 ± 0.009 | 0.965 ± 0.021 |
|  |  |  |  |  |
| 10^th^ Percentile training presence | AUC | 0.762 ± 0.003 | 0.892 ± 0.075 | 0.835 ± 0.044 |
|  | Threshold | 0.630 ± 0.001 | 0.477 ± 0.344 | 0.135 ± 0.062 |
|  | Sensitivity | 0.900 ± 0.005 | 0.833 ± 0.192 | 0.708 ± 0.083 |
|  | Specificity | 0.624 ± 0.003 | 0.950 ± 0.054 | 0.962 ± 0.008 |
|  | Omission rate | 0.100 ± 0.005 | 0.167 ± 0.192 | 0.292 ± 0.083 |
|  | Proportion correct | 0.661 ± 0.002 | 0.950 ± 0.054 | 0.962 ± 0.008 |
|  | Fractional predicted area | 0.376 ± 0.003 | 0.050 ± 0.054 | 0.038 ± 0.008 |
|  | Kappa | 0.263 ± 0.003 | 0.046 ± 0.049 | 0.030 ± 0.009 |
|  | TSS | 0.524 ± 0.006 | 0.783 ± 0.150 | 0.670 ± 0.088 |
|  |  |  |  |  |
| Maximum test sensitivity plus specificity | AUC | 0.767 ± 0.005 | 0.900 ± 0.092 | 0.873 ± 0.043 |
|  | Threshold | 0.638 ± 0.007 | 0.457 ± 0.235 | 0.111 ± 0.086 |
|  | Sensitivity | 0.883 ± 0.018 | 0.833 ± 0.192 | 0.792 ± 0.083 |
|  | Specificity | 0.651 ± 0.023 | 0.966 ± 0.017 | 0.954 ± 0.007 |
|  | Omission rate | 0.117 ± 0.018 | 0.167 ± 0.192 | 0.208 ± 0.083 |
|  | Proportion correct | 0.682 ± 0.018 | 0.966 ± 0.017 | 0.954 ± 0.008 |
|  | Fractional predicted area | 0.349 ± 0.023 | 0.034 ± 0.017 | 0.046 ± 0.007 |
|  | Kappa | 0.280 ± 0.014 | 0.037 ± 0.032 | 0.028 ± 0.007 |
|  | TSS | 0.534 ± 0.010 | 0.800 ± 0.184 | 0.746 ± 0.086 |

| **Region** | **Current** | **SD** | **Scenario** | **Prob** | **SD** | **% change** | **Paired t** | **df** | **p** |
| --- | --- | --- | --- | --- | --- | --- | --- | --- | --- |
|  |  |  |  |  |  |  |  |  |  |
| **a) Europe_Worldclim_** | | | |  |  |  |  |  |  |
|  |  |  |  |  |  |  |  |  |  |
| Ireland | 0.653 | 0.017 | 2050 (4.5) | 0.664 | 0.017 | 1.7 | 175.0 | 7,036 | <0.001 |
|  |  |  | 2050 (8.5) | 0.655 | 0.017 | 0.3 | 18.1 | 7,036 | <0.001 |
|  |  |  | 2070 (4.5) | 0.659 | 0.017 | 0.9 | 70.6 | 7,036 | <0.001 |
|  |  |  | 2070 (8.5) | 0.664 | 0.017 | 1.6 | 83.8 | 7,036 | <0.001 |
|  |  |  |  |  |  |  |  |  |  |
| Great Britain | 0.636 | 0.042 | 2050 (4.5) | 0.660 | 0.043 | 3.9 | 431.8 | 19,807 | <0.001 |
|  |  |  | 2050 (8.5) | 0.658 | 0.048 | 3.5 | 248.9 | 19,807 | <0.001 |
|  |  |  | 2070 (4.5) | 0.659 | 0.046 | 3.6 | 308.4 | 19,807 | <0.001 |
|  |  |  | 2070 (8.5) | 0.665 | 0.054 | 4.6 | 241.8 | 19,807 | <0.001 |
|  |  |  |  |  |  |  |  |  |  |
| Europe | 0.622 | 0.084 | 2050 (4.5) | 0.678 | 0.054 | 8.9 | 755.7 | 277,597 | <0.001 |
|  |  |  | 2050 (8.5) | 0.683 | 0.048 | 9.7 | 684.0 | 277,597 | <0.001 |
|  |  |  | 2070 (4.5) | 0.684 | 0.047 | 9.9 | 684.7 | 277,597 | <0.001 |
|  |  |  | 2070 (8.5) | 0.700 | 0.046 | 12.5 | 742.9 | 277,597 | <0.001 |
|  |  |  |  |  |  |  |  |  |  |
| Scandinavia | 0.330 | 0.120 | 2050 (4.5) | 0.460 | 0.120 | 38.0 | 1083.1 | 122,845 | <0.001 |
|  |  |  | 2050 (8.5) | 0.500 | 0.120 | 50.8 | 1078.4 | 122,845 | <0.001 |
|  |  |  | 2070 (4.5) | 0.480 | 0.120 | 45.3 | 1095.7 | 122,845 | <0.001 |
|  |  |  | 2070 (8.5) | 0.510 | 0.120 | 54.1 | 953.2 | 122,845 | <0.001 |
|  |  |  |  |  |  |  |  |  |  |
| Baltic | 0.452 | 0.035 | 2050 (4.5) | 0.589 | 0.020 | 30.2 | 885.6 | 15,455 | <0.001 |
|  |  |  | 2050 (8.5) | 0.613 | 0.017 | 35.6 | 934.3 | 15,455 | <0.001 |
|  |  |  | 2070 (4.5) | 0.601 | 0.020 | 33.0 | 870.0 | 15,455 | <0.001 |
|  |  |  | 2070 (8.5) | 0.629 | 0.019 | 39.1 | 878.1 | 15,455 | <0.001 |
|  |  |  |  |  |  |  |  |  |  |
| **b) Ireland_Worldclim_** | | | |  |  |  |  |  |  |
|  |  |  |  |  |  |  |  |  |  |
| Ireland | 0.020 | 0.090 | 2050 (4.5) | 0.140 | 0.300 | 534.8 | 41.6 | 7,044 | <0.001 |
|  |  |  | 2050 (8.5) | 0.180 | 0.300 | 721.8 | 49.9 | 7,044 | <0.001 |
|  |  |  | 2070 (4.5) | 0.190 | 0.300 | 734.8 | 48.2 | 7,044 | <0.001 |
|  |  |  | 2070 (8.5) | 0.260 | 0.400 | 1049.6 | 53.3 | 7,044 | <0.001 |
|  |  |  |  |  |  |  |  |  |  |
| **c) Ireland_ICHEC_ - All Ireland** | | | |  |  |  |  |  |  |
|  |  |  |  |  |  |  |  |  |  |
| Ireland | 0.045 | 0.138 | 2050 (4.5) | 0.133 | 0.272 | 195.6 | 37.7 | 5,490 | <0.001 |
|  |  |  | 2050 (8.5) | 0.142 | 0.283 | 215.6 | 38.2 | 5,490 | <0.001 |
|  |  |  | 2070 (4.5) | 0.154 | 0.301 | 242.2 | 38.4 | 5,490 | <0.001 |
|  |  |  | 2070 (8.5) | 0.167 | 0.310 | 271.1 | 40.8 | 5,490 | <0.001 |
|  |  |  |  |  |  |  |  |  |  |
| **d) Ireland_ICHEC_ - Natterjack occupied cells only** | | | | | | | | | |
|  |  |  |  |  |  |  |  |  |  |
| Ireland | 0.787 | 0.215 | 2050 (4.5) | 0.992 | 0.014 | 26.0 | 3.4 | 11 | 0.005 |
|  |  |  | 2050 (8.5) | 0.998 | 0.003 | 26.8 | 3.4 | 11 | 0.006 |
|  |  |  | 2070 (4.5) | 0.995 | 0.007 | 26.4 | 3.4 | 11 | 0.006 |
|  |  |  | 2070 (8.5) | 0.999 | 0.002 | 26.9 | 3.4 | 11 | 0.006 |

**Table S3** Paired t-tests (paired at the level of the grid cell) for change in predicted suitability between current conditions and future climate scenarios for **a)** Europe using WorldClim (separated into regions), **b)** Ireland using WorldClim **c)** Ireland using ICHEC climate data, **d)** Ireland using ICHEC climate data and only those cells occupied by toads.

**Table S4** 2x2 χ^2^ contingency tests of association for change in bioclimatic envelope (number of suitable cells above the MaxTSS threshold) for **a)** Europe using Worldclim (separated into regions), **b)** Ireland using Worldclim and **c)** Ireland using ICHEC climate data.

| **Region** | ***n* (cells)** | **Baseline** | **% suitable** | **% unsuitable** |  | **Future scenario** | **% suitable** |  | **χ^2^** | **df** | ***p*** |  |
| --- | --- | --- | --- | --- | --- | --- | --- | --- | --- | --- | --- | --- |
| **a)       Europe_Worldclim_** | | | | | | | | | | | | |
| Ireland | 7,037 | Current | 100 | 0 |  | 2050 (4.5) | 100 |  | *na* | 1 | 1 |  |
|  |  |  |  |  |  | 2050 (8.5) | 100 |  | *na* | 1 | 1 |  |
|  |  |  |  |  |  | 2070 (4.5) | 100 |  | *na* | 1 | 1 |  |
|  |  |  |  |  |  | 2070 (8.5) | 100 |  | *na* | 1 | 1 |  |
|  |  |  |  |  |  |  |  |  |  |  |  |  |
| Great Britain | 19,808 | Current | 99.4 | 0.6 |  | 2050 (4.5) | 100 |  | 104 | 1 | <0.001 |  |
|  |  |  |  |  |  | 2050 (8.5) | 99.6 |  | 9 | 1 | 0.003 |  |
|  |  |  |  |  |  | 2070 (4.5) | 100 |  | 85 | 1 | <0.001 |  |
|  |  |  |  |  |  | 2070 (8.5) | 99.7 |  | 17 | 1 | <0.001 |  |
|  |  |  |  |  |  |  |  |  |  |  |  |  |
| Europe | 277,598 | Current | 90.9 | 9.1 |  | 2050 (4.5) | 98.5 |  | 16,038 | 1 | <0.001 |  |
|  |  |  |  |  |  | 2050 (8.5) | 99.4 |  | 21,366 | 1 | <0.001 |  |
|  |  |  |  |  |  | 2070 (4.5) | 99.3 |  | 20,967 | 1 | <0.001 |  |
|  |  |  |  |  |  | 2070 (8.5) | 99.4 |  | 21,978 | 1 | <0.001 |  |
|  |  |  |  |  |  |  |  |  |  |  |  |  |
| Scandinavia | 122,846 | Current | 13.2 | 86.8 |  | 2050 (4.5) | 37.1 |  | 18,666 | 1 | <0.001 |  |
|  |  |  |  |  |  | 2050 (8.5) | 51.8 |  | 41,792 | 1 | <0.001 |  |
|  |  |  |  |  |  | 2070 (4.5) | 45.9 |  | 31,561 | 1 | <0.001 |  |
|  |  |  |  |  |  | 2070 (8.5) | 55.6 |  | 48,952 | 1 | <0.001 |  |
|  |  |  |  |  |  |  |  |  |  |  |  |  |
| Baltic | 15,456 | Current | 7.3 | 92.7 |  | 2050 (4.5) | 100 |  | *Inf* | 1 | <0.001 |  |
|  |  |  |  |  |  | 2050 (8.5) | 100 |  | *Inf* | 1 | <0.001 |  |
|  |  |  |  |  |  | 2070 (4.5) | 100 |  | *Inf* | 1 | <0.001 |  |
|  |  |  |  |  |  | 2070 (8.5) | 100 |  | *Inf* | 1 | <0.001 |  |
|  |  |  |  |  |  |  |  |  |  |  |  |  |
| **b)       Ireland_Worldclim_** | 7,045 | Current | 16.3 | 83.7 |  | 2050 (4.5) | 31.4 |  | 1750 | 1 | <0.001 |  |
|  |  |  |  |  |  | 2050 (8.5) | 37.5 |  | 1670 | 1 | <0.001 |  |
|  |  |  |  |  |  | 2070 (4.5) | 37.7 |  | 1690 | 1 | <0.001 |  |
|  |  |  |  |  |  | 2070 (8.5) | 41.8 |  | 2070 | 1 | <0.001 |  |
|  |  |  |  |  |  |  |  |  |  |  |  |  |
| **c)       Ireland_ICHEC_** | 5,491 | Current | 3 | 97 |  | 2050 (4.5) | 11.1 |  | 232 | 1 | <0.001 |  |
|  |  |  |  |  |  | 2050 (8.5) | 12 |  | 280 | 1 | <0.001 |  |
|  |  |  |  |  |  | 2070 (4.5) | 13.6 |  | 359 | 1 | <0.001 |  |
|  |  |  |  |  |  | 2070 (8.5) | 14.9 |  | 429 | 1 | <0.001 |  |

|  | **% change** | | |
| --- | --- | --- | --- |
|  | **Suitability**  (predicted probability) |  | **Bioclimatic envelope**  (potentially suitable cells) |
| **a) Europe_Worldclim_** | 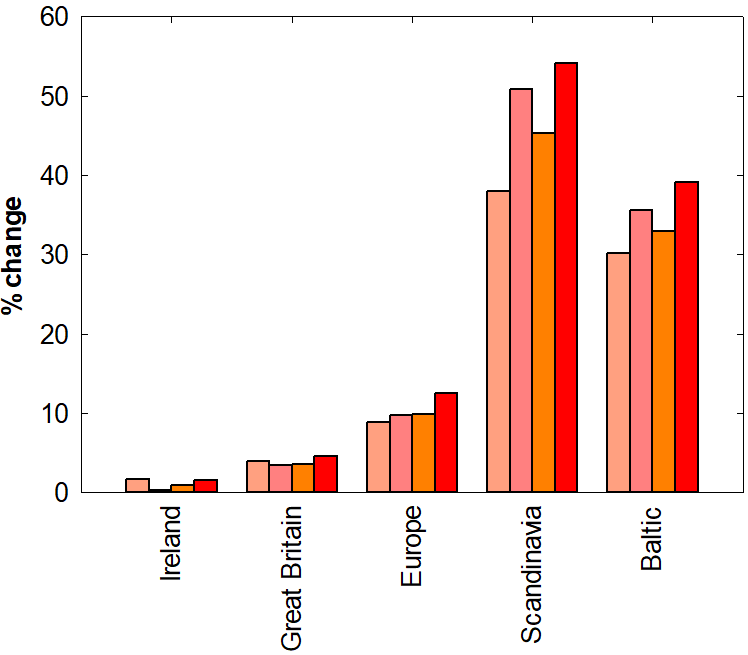 |  | 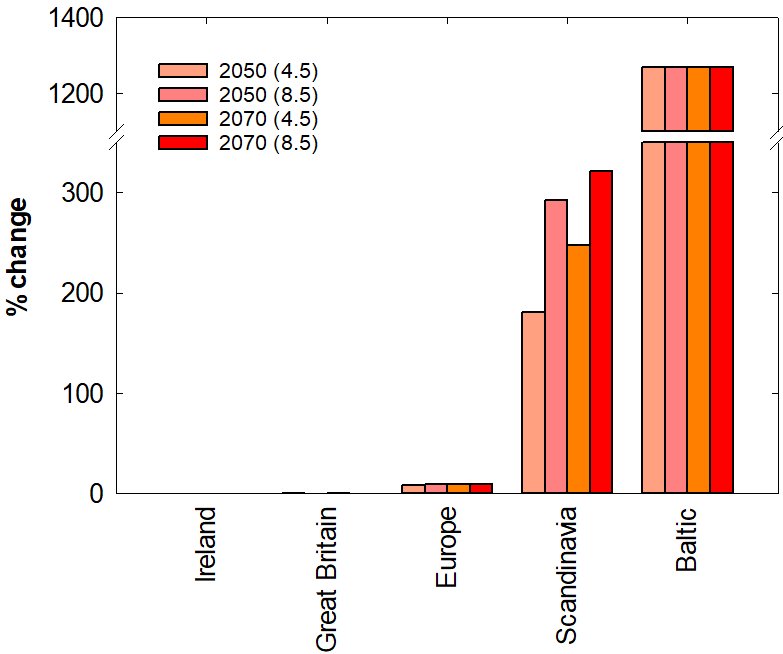 |
| **b) Ireland_Worldclim_** | 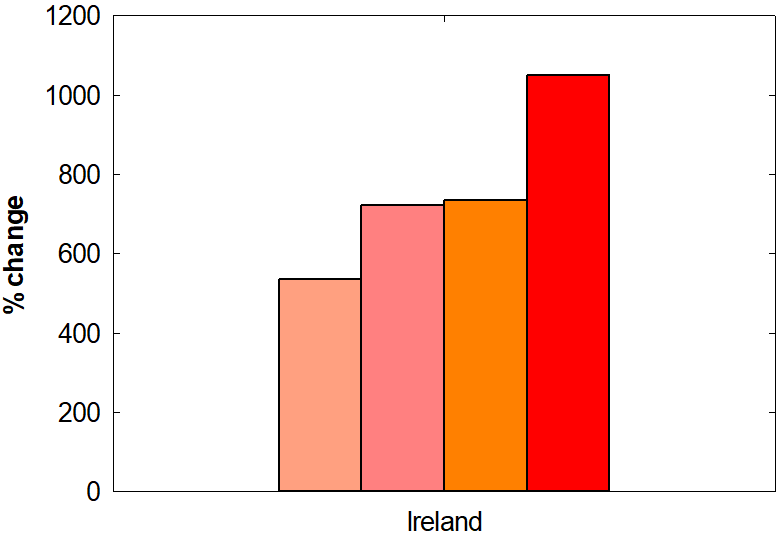 |  | 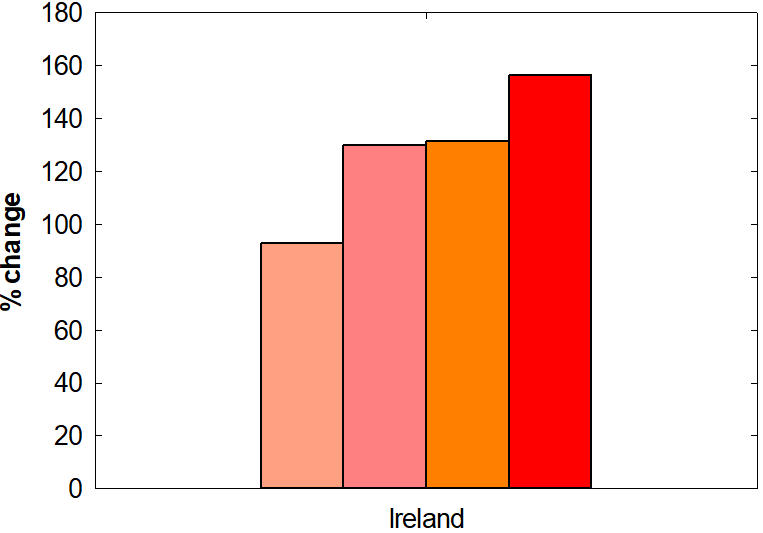 |
| **c) Ireland_ICHEC_** | 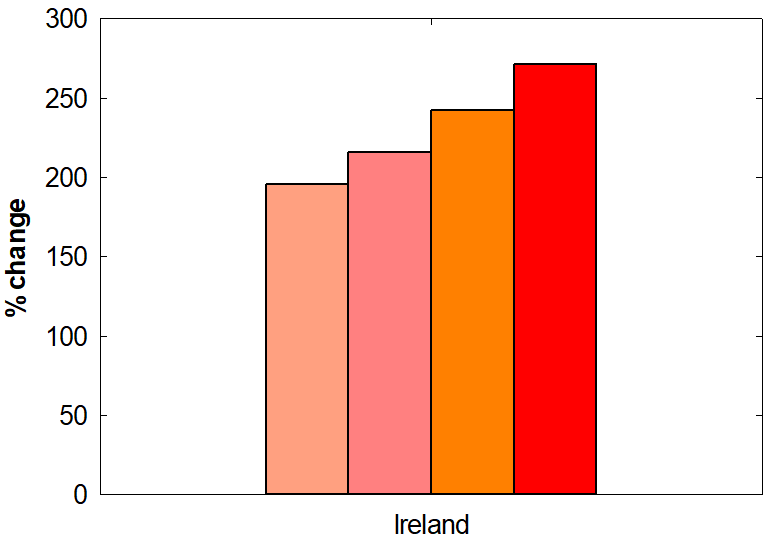 |  | 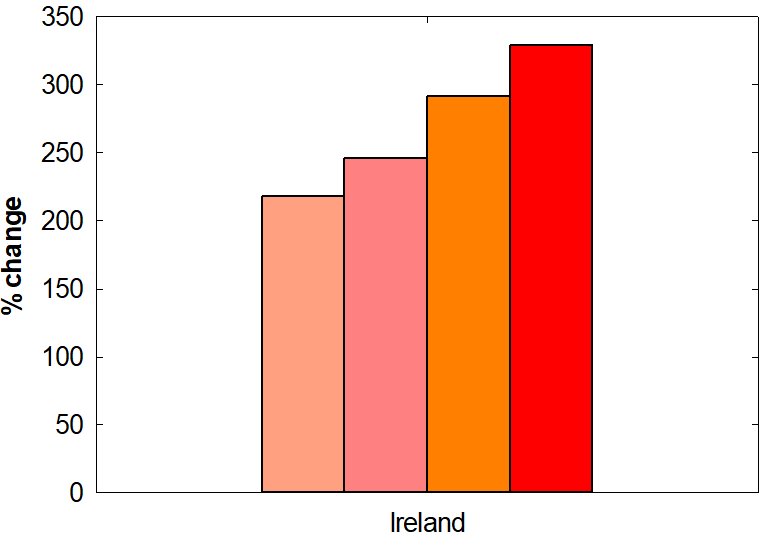 |

**Figure S3** Predicted percentage (%) change in suitability (left column) and potential suitable cells (right column) for Natterjack toads in **a)** Europe using Worldclim (separated into regions), **b)** Ireland using Worldclim and **c)** Ireland using ICHEC climate data between current conditions and future climate change scenarios (bars from left-to-right). Note *y*-axes values are not standardized due to the large range between regions and models.
